# Siloxane-PEO-PPO Hybrid Materials Containing Superparamagnetic Iron Oxide Nanoparticles: Effect of Particle Surface Functionalization on the Structure and Hyperthermia Properties

**DOI:** 10.1021/acsomega.6c01026

**Published:** 2026-06-06

**Authors:** Agnes Candido Teixeira, Natasha Midori Suguihiro, Benjamin Rache Salles, Pedro Carvalho Ramos, Luiz Augusto Sousa de Oliveira, Karim Dahmouche

**Affiliations:** † Campus de Duque de Caxias, Universidade Federal do Rio de Janeiro (UFRJ), Duque de Caxias, RJ, Brazil CEP 25240-000; ‡ Instituto de Física, 28125 Universidade Federal do Rio de Janeiro (UFRJ), Rio de Janeiro, RJ, Brazil CEP 21941-909

## Abstract

This study explores the development and characterization of hybrid siloxane-polyether nanocomposites incorporating superparamagnetic iron oxide nanoparticles for potential applications requiring magnetic hyperthermia. The effect of the hydrophilic or hydrophobic surface functionalization of the nanoparticles on the structural features, magnetic properties, and hyperthermia performance of a siloxane-poly­(ethylene oxide) (PEO)–poly­(propylene oxide) (PPO) hybrid matrix has been investigated. X-ray diffraction (XRD) and Fourier transform infrared spectroscopy (FTIR) measurements revealed the successful synthesis of both the expected superparamagnetic iron oxide phases and the hybrid material, without compromising the structural integrity of the hybrid matrix. Scanning electron microscopy combined with energy dispersive X-ray spectroscopy (SEM/EDS) showed aggregated iron oxide nanoparticles in the matrix forming microsized clusters. In contrast, small-angle X-ray scattering (SAXS) revealed the presence of well-dispersed iron oxide nanoparticles between the clusters, in agreement with particle–matrix interactions detected by FTIR and XRD. Magnetic characterization using a vibrating sample magnetometer (VSM) revealed that hybrids loaded with hydrophobic nanoparticles display a lower superparamagnetic behavior, and hyperthermia tests showed a reduced performance inside the hybrid matrix compared to hydrophilic nanoparticles. This reduction is due to the effective loss of particle coating when the nanoparticles are inserted into the hybrid matrix and a lower amount of organic coating (as detected by thermogravimetric analysis (TGA)), leading to a decrease in the magnetic moment and increased nanoparticle aggregation. These results confirm the importance of adequate control of nanoparticle surface chemistry for future optimization of hyperthermia properties of these promising flexible and biocompatible hybrid materials for innovative magnetic systems.

## Graphical abstract

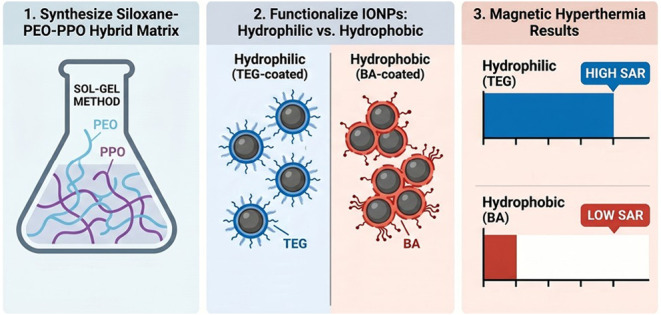

## Introduction

Magnetic hyperthermia (MHT) has emerged as a minimally invasive therapeutic modality that employs magnetic nanoparticles (MNPs) to generate localized heat upon exposure to an external alternating magnetic field (AMF).^1,2^ The conversion of magnetic energy into thermal energy elevates the temperature in the target tissue to a mild hyperthermic range, typically 42–46 °C,^2–4^ directly triggering apoptotic cell death or sensitizing pathological tissues to conventional treatments.^3,5^ MHT scope includes theranostic platforms with magnetic particle imaging (MPI) for guided drug delivery, tissue regeneration, and antibacterial treatments.^2,5–7^ Despite its promise, clinical translation is hindered by suboptimal heating efficiency, quantified by the Specific Absorption Rate (SAR) and nanoparticle agglomeration.^4,8,9^ To address these limitations, polymeric nanocomposites offer a strategic platform by encapsulating MNPs to improve their colloidal stability and biocompatibility.^3,10^

In this context, organic–inorganic hybrid (OIH) materials,^11,12^ specifically Siloxane-Poly­(ethylene oxide) (PEO) and Siloxane-Poly­(propylene oxide) (PPO) synthesized by the sol–gel method, emerge as innovative candidates, providing enhanced mechanical and thermal properties, over conventional purely organic polymers.^13^ Furthermore, recent advancements highlight that the spatial confinement of IONPs within solid matrices is highly effective at preventing agglomeration and enhancing magnetothermal responses.^14^ These hybrids are currently under investigation for their potential in biological applications^15–19^ and are well-known as ureasils, for being a siloxane framework covalently bonded to polyether chains through urea linkages.^20^ They can be engineered into thin films, yielding transparent, biocompatible, and flexible materials.^16^ Matrices derived from PEO are particularly valued for the polymer’s low toxicity and high swelling, making them effective matrices for dissolving ionic species and polar molecules leading to their biological exploration.^15–19^ Conversely, Siloxane-PPO hybrids share many advantages but exhibit a more hydrophobic character.^15,16^

However, despite the enhanced properties of OIH over conventional polymers, its application in the design of superparamagnetic nanocomposites for innovative magnetic hyperthermia-based devices remains underexplored. To impart this superparamagnetic character, it is possible to use iron oxide nanoparticles (IONPs), which are known for their nontoxicity, biocompatibility,^21–25^ and exhibit superparamagnetic behavior, showing high heat-generating capacity under an alternating magnetic field, easy preparation, and facile chemical functionalization.^21–24^ There has been limited research in making OIH responsive to magnetic stimuli by incorporating IONPs. Investigations combining ureasil polyether-based materials with superparamagnetic IONPs are even scarcer,^26,27^ despite these IONPs being the most extensively studied inorganic nanocarrier systems for drug delivery applications.^21,22^ Previous studies have shown that incorporating IONPs^26^ or CoFe_2_O_4_ ^27^ in Siloxane-PEO or Siloxane-PPO hybrids stimulates drug release through matrix thermal expansion and polymer melting, and highlighted that PPO-based ureasils present superior hyperthermia efficiency due to their lower heat capacity. Consequently, developing a Siloxane-PPO-PEO hybrid blend (70 wt % PPO_400_ and 30 wt % PEO_800_) is proposed as a pertinent strategy to combine the superior magnetic hyperthermia efficiency and low nanoparticle leaching of PPO with the improved swellability, drug release kinetics, and bioadhesion of PEO. Furthermore, the hydrophobic character of PPO results in slow sustained drug release in aqueous media such as biological environments due to the very low water diffusion kinetic and swelling in this polymer.^27–29^ The inclusion of a 30% hydrophilic PEO component also aims to modulate this release profile, as blending PEO and PPO has been proven to be an effective strategy for fine-tuning the material’s swellability and drug release kinetics.^28,29^

To ensure optimal heat transfer and prevent nanoparticle aggregation within this specific matrix, it is possible to control the hydrophobicity of the IONPs surface^21–23^ by surface functionalization, which consists of coating the particles to improve their colloidal stability^30,31^ preventing agglomeration through steric interactions and electrostatic repulsions between the particles.^30,32^ Indeed, recent studies emphasize that tailoring the surface from a hydrophobic to a hydrophilic nature is a critical step to ensure colloidal stability and prevent magnetic dipole–dipole attractions in complex matrices.^33,34^ A strategic approach to achieve this is the use of nonaqueous synthesis routes, such as microwave-assisted solvothermal polyol methods using benzyl alcohol (BA) or triethylene glycol (TEG), which directly produce highly crystalline, uniform nanoparticles with specifically tailored hydrophobic or hydrophilic surfaces.^30,35,36^

Therefore, to fill the existing literature gap, this work aims to investigate how the hydrophilic (TEG-functionalized) or hydrophobic (BA-functionalized) nature of IONPs incorporated into an OIH Siloxane-PEO-PPO matrix affects the structure, magnetic properties, and hyperthermia performance of the resulting nanocomposites. Because the hybrid matrix is predominantly hydrophobic (70 wt % PPO), investigating BA-functionalized IONPs provides a fundamental model to test whether matching the hydrophobic character of the nanoparticles to the matrix enhances structural dispersion, whereas TEG-functionalized IONPs serve as a hydrophilic counter-model to evaluate the role of contrasting surface affinities. The morphological and nanostructural features of the hybrid nanocomposites, as well as their magnetic properties and hyperthermia behavior, were comprehensively evaluated by X-ray diffraction (XRD), Fourier Transform Infrared Spectroscopy (FTIR), Scanning Electron Microscopy (SEM), Energy Dispersive X-ray Spectroscopy (EDS), Small-Angle X-ray Scattering (SAXS), Vibrating Sample Magnetometry (VSM), and magnetic hyperthermia (MH) measurements. The Supporting Information also presents some of the results related to the iron oxide (γ-Fe_2_O_3_ or Fe_3_O_4_) nanoparticles.

## Materials and Methods

### Materials

Iron­(III) acetylacetonate (Fe­(C_5_H_7_O_2_)_3_, CAS no. 14024-18-1), triethylene glycol (HO­(CH_2_CH_2_O)_2_CH_2_CH_2_OH, CAS no. 112-27-6), benzyl alcohol (C_6_H_5_CH_2_OH, CAS no. 100-51-6), poly­(propylene glycol) bis (2-aminopropyl ether) (CH_3_CH­(NH_2_)­CH_2_[OCH_2_CH­(CH_3_)]_n_NH_2_, CAS no. 9046-10-0), *O*,*O*′-Bis­(2-aminopropyl) polypropylene glycol-*block*-polyethylene glycol-*block*- polypropylene glycol (CH_3_CH­(NH_2_)­CH_2_[OCH­(CH_3_)­CH_2_]_l_(OCH_2_CH_2_)_m_[OCH_2_CH­(CH_3_)]_n_NH_2_, CAS no. 65605-36-9), 3-(Triethoxysilyl)­propyl isocyanate ((C_2_H_5_O)_3_Si­(CH_2_)_3_NCO, CAS no. 24801-88-5) and tetrahydrofuran (C_4_H_8_O, CAS no. 109-99-9) were purchased from Sigma-Aldrich. Ethyl alcohol (C_2_H_5_OH, CAS no. 64-17-5) was purchased from Neon. Water used in the experiments was ultrapure (Type 1), produced using a Direct-QR 3 UV system from Millipore (Merck).

### Synthesis and Functionalization of Iron Oxide Nanoparticles

IONPs were synthesized using a solvothermal approach described in the referenced studies.^35–37^ Parameters such as temperature (*T*) and molar concentration of the precursor (*X*) were set to 200 °C and 2.8 mmol of iron­(III) acetylacetonate (Fe­(acac)_3_) per 20 mL of Triethylene glycol (TEG), which was used to produce hydrophilic nanoparticles, whereas Benzyl alcohol (BA) was employed to synthesize hydrophobic nanoparticles, following established literature protocols. The synthesis time was adjusted between 1 and 4 h. A reaction mixture placed in a glass vessel was introduced into the microwave reactor, which was equipped with a cylindrical magnetic stir bar. The system was programmed for rapid heating to the desired synthesis temperature, with a stirring speed of 600 rpm and a cooling temperature of 55 °C postsynthesis. Targeting a 100% theoretical conversion of Fe­(acac)_3_, the process is expected to yield a colloidal solution with either Fe_3_O_4_ (magnetite) and/or γ-Fe_2_O_3_ (maghemite) concentration of 11.4 mg/mL.

### Synthesis of Ureasil-Polyether Precursors

The synthesis of hybrid precursors followed a standardized procedure.^39^ Urea groups connecting silicon atoms and the polyether chains were formed by reaction between the aminopropyl terminal groups of functionalized poly­(ethylene oxide) (PEO) (*O*,*O*-bis­(2-aminopropyl)-poly­(ethylene oxide)) or poly­(propylene oxide) (PPO) (*O*,*O*-bis­(2-aminopropyl)-poly­(propylene oxide)) with 3-(isocyanatopropyl)-triethoxysilane in a molar ratio of 1:2. The mixture was stirred in tetrahydrofuran (THF) under reflux conditions for 24 h. The THF was then removed through evaporation at 80 °C, resulting in the formation of a hybrid precursor presenting covalent bonds between the inorganic and organic parts. Poly­(ethylene oxide) (PEO) with an average molecular weight (*M*_w_) of 800 g mol^–1^ and poly­(propylene oxide) (PPO) with an average molecular weight (*M*_w_) of 400 g mol^–1^ were used as shown in Scheme 1.

**Scheme 1:**
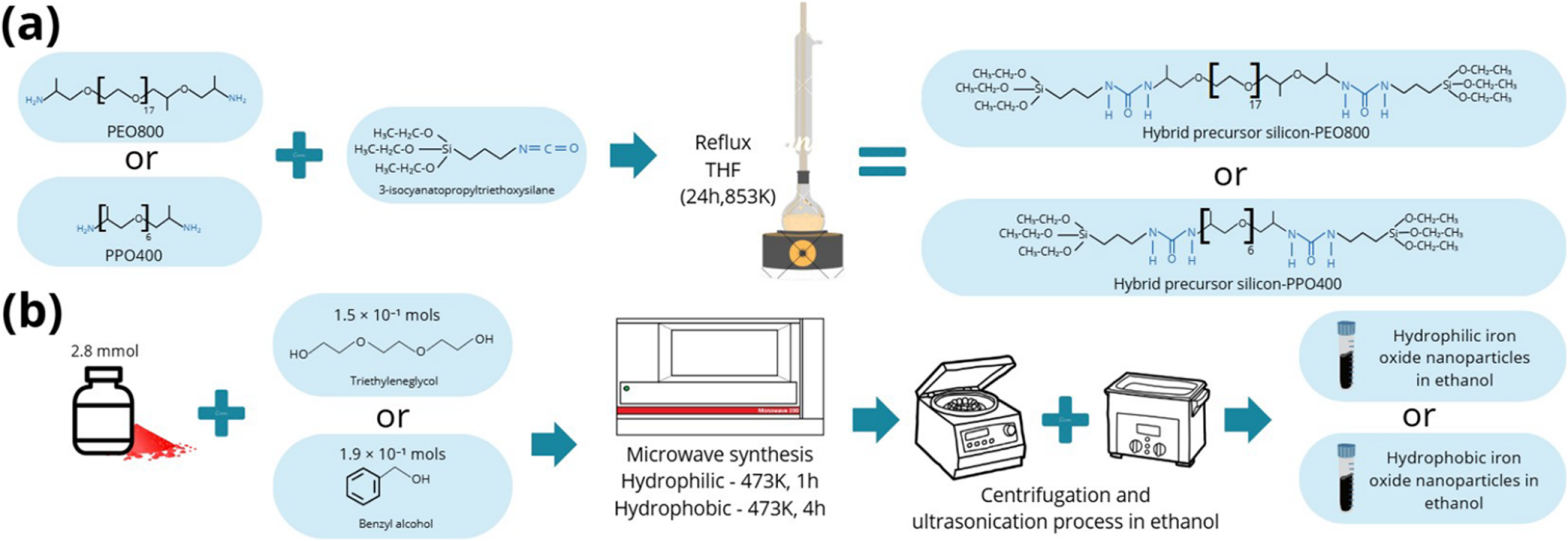
(a) Preparation of Hybrid Polymeric Precursors, and (b) Schematic Representation of the Synthesis Procedure for Hydrophilic and Hydrophobic Nanoparticles

### Synthesis of Nanocomposites

0.35 g of the previously synthesized silicon-PPO_400_ precursor and 0.15 g of the silicon-PEO_800_ precursor were blended with 2.3 mL of the colloidal suspension of IONPs dispersed in ethanol. After calculating the actual amounts of PPO and PEO in each precursor, taking into account the PPO segments present in the silicon-PEO_800_ precursor and the terminal groups, the resulting mixture corresponds to approximately 69 wt % PPO and 31 wt % PEO in the polymer phase. Furthermore, the amount of IONPs was intended to correspond to 5% of the mass of the hybrid polymer to be used. However, by the end of the preparation process, a change in the final proportion of nanoparticles relative to the matrix was observed due to the presence of functional groups on their surface and material loss during the procedure. For this reason, the actual concentration of IONP mass was determined through TGA/DSC experiments, taking into account the polymer degradation and measuring the residual IONPs mass. This approach allowed for the synthesis of nanocomposites with IONP concentrations ranging from 15 to 18%, after subtracting the contributions of water, ethanol, and organic ligands on the particle surfaces. The mixture was then left to stand at room temperature under stirring for 10 min. Subsequently, water was added while stirring to initiate the hydrolysis and polycondensation reactions of the hybrid precursor. The volume of water added was dependent on the chain length and was introduced at a ratio of 30 times greater than required to enhance hydrolysis. The wet gel formation proceeded through the hydrolysis of −(SiOEt_3_)_3_ groups, followed by a condensation reaction that created the siloxane cross-linked nodes between PEO or PPO chains. After gelation, the samples were placed in a cylindrical mold (approximately 20 mm in diameter) for 72 h for drying under vacuum at 60 °C, forming solid disks with a thickness of approximately 1 mm. The resulting siloxane-polyether solid nanocomposite disks were designated as siloxane-PPO-PEO-IONPs (70:30 PPO:PEO). No phase separation between PEO and PPO polymers was observed due to the presence of the siloxane nodes acting as cross-linkers between both types of polymer chains.^28,29^

### Characterization Techniques

To determine the hydrodynamic size distribution, polydispersity, and colloidal state of the synthesized nanoparticles, Dynamic Light Scattering (DLS) analysis was performed. Because hydrophilic and hydrophobic nanoparticles possess opposite surface affinities, they cannot be stably dispersed in the same solvent. Therefore, to minimize precipitation during the measurements, the nanoparticles were dispersed in solvents compatible with their respective coatings. The hydrophilic nanoparticles were analyzed in water, while the hydrophobic nanoparticles were dispersed in an organic solvent. The concentration was adjusted to ensure the nanoparticle dispersion had a transmittance of approximately 70%. Prior to analysis, all dilutions were treated with an ultrasonic bath for 10 to 20 min. The diluted samples were then placed in disposable cuvettes and introduced into the Litesizer 500 instrument from Anton Paar. The Dynamic Light Scattering (DLS) analysis for both hydrophilic and hydrophobic nanoparticles used similar materials and common settings but exhibited notable differences in specific conditions and solvents. For both types of nanoparticles, the material analyzed was selected as hematite (Fe_2_O_3_), with a refractive index of 3.0510 and an absorption coefficient of 0.1000. The target temperature for both analyses was maintained at 25 °C, with an equilibration time of 30 s. The analysis model employed was the general model, along with an advanced cumulant model. The focus position was set to 0 mm automatically in both cases.

For transmission Electron Microscopy (TEM) of the IONPs, a solution consisting of 5 mL of isopropyl alcohol and 20 μL of the colloidal solution containing the nanoparticles was prepared. This procedure was performed for both hydrophilic and hydrophobic nanoparticles. The resultant mixtures were then subjected to ultrasonication for 20 min. Drops of these solutions were carefully deposited onto lacey carbon Cu-grids for subsequent analysis. The analysis was performed using a JEOL JEM 2100F, operated at 200 kV in phase contrast mode. Nanoparticle size measurements were conducted using Gatan Digital Micrograph software, with data processing to generate histogram curves performed in Origin 2024 software.

To identify the crystalline phases and confirm the structural integrity of the synthesized iron oxide cores, X-ray Diffraction (XRD) of the nanoparticles was performed. Prior to analysis, the samples underwent a washing process involving centrifugation and ultrasonication cycles. The sample was centrifuged at 3600 rpm for 10 min, and the supernatant was discarded. The pellet was resuspended in water, and this cycle was repeated four times. A portion of the washed particles was used for hyperthermia experiments. The remaining particles were lyophilized and subsequently used for XRD analysis, as well as for hyperthermia testing after being resuspended in water at a concentration of 10 mg/mL. X-ray Diffraction (XRD) of nanoparticles was performed using an Empyrean diffractometer system. The measurements were conducted with Cu Kα radiation (λ = 0.15406 nm) at a voltage of 40 kV and a current of 40 mA. The diffractometer was configured in a Bragg–Brentano Theta/2Theta geometry (PW3050/60), and the sample stage used was a Reflection-Transmission Spinner (PW3064/60). The analysis was carried out in reflection mode, with a continuous scan mode along the goniometer axis. The diffraction patterns were recorded over a 2θ range of 10.0 to 80.0° with a minimum step size of 0.012°. Data collection was performed using a PIXcel3D detector in line scanning mode, and the spinner revolution time was set to 2 s per revolution to ensure homogeneity in data collection.

To investigate the structural organization and the interchain periodicity of the hybrid matrices, X-ray diffraction (XRD) experiments of the nanocomposites were conducted using a Bruker D8 Discover diffractometer with Cu Kα radiation (λ = 0.15406 nm) at a voltage of 40 kV and a current of 40 mA. The measurements were performed over a 2θ range from 5 to 70°, with a step size set of 0.02° per second. Background subtraction and data analysis were carried out using the FullProf software and Origin 2024, comparing the obtained diffraction patterns with reference data from the literature to identify the crystalline phases in the samples. The solid nanocomposite disks were analyzed in their intact form without any grinding.

Fourier Transform Infrared Spectroscopy (FTIR) of the nanocomposites using the Attenuated Total Reflectance (ATR) method was performed to investigate particle–matrix interactions. The measurements were carried out using an IRSpirit spectrometer equipped with Shimadzu’s QATR-S ATR accessory. The analysis was performed on both the neat hybrid matrix and the nanocomposite containing IONPs. Spectra were recorded in the range of 4000 to 400 cm^–1^ with a resolution of 1.5 cm^–1^. Data analysis was performed using Origin 2024 software, comparing the obtained spectra with reference spectra from the literature.

Scanning electron microscopy (SEM) was employed to examine the surface morphology of the solid nanocomposite disks. The nanocomposite samples were mounted on aluminum stubs by using conductive carbon tape. The samples were then sputter-coated with a thin carbon layer to enhance the conductivity and image quality. Analyses were performed using a JEOL JSM-IT700HR microscope, operated with an acceleration voltage of 10 to 15 kV and backscattered electron imaging. Data analysis was performed using digital microscopy software, comparing the obtained images with literature reference data to evaluate the dispersion and distribution of nanoparticles within the solid nanocomposite disks.

The nanostructural features of the nanocomposites were investigated by Small-Angle X-ray Scattering (SAXS). The SAXS experiments were carried out with the Nanostar SAXS camera from Bruker AXS, using 40 kV and 35 mA. A position-sensitive X-ray detector PILATUS was used to record the SAXS intensity. The scattering intensity *I*(*q*) was plotted as a function of the modulus of the scattering vector $q=\frac{4\pi \;\sin\theta }{\lambda }$ , where θ is the scattering angle and λ = 1.54 nm. SAXS data processing was performed with the software OriginPro 8.0 and involved fitting of the experimental curves at low *q*-range using the Guinier equation:^38^

1 $$I\left(q\right)=I\left(0\right)\exp\left(-\frac{q^{2}{R_{g}}^{2}}{3}\right)$$

where *R*_g_ is the radius of gyration of nanoparticles and *I*(0) depends on the number of nanoparticles (*N*), the electron density contrast (ρ_p_ – ρ_m_) between nanoparticles and matrix, and the particle volume (*v*). The fit of the experimental SAXS curves at low q-range by the theoretical function described in eq 1 was performed by the least-squares method.

The average distance between siloxane nodes (*d*) was calculated through the equation:^38^

2 $$d=\frac{2\pi }{q_{\max}}$$

*q*_max_ is the modulus of the scattering vector at the peak maximum.

For Fourier Transform Infrared Spectroscopy (FTIR), Scanning Electron Microscopy/Energy Dispersive X-ray Spectroscopy (SEM/EDS), and Small-Angle X-ray Scattering (SAXS), a piece of the solid nanocomposite disks was cut and placed in an oven for drying at 333 K (60 °C). After drying, the solid nanocomposite disks were divided into separate pieces for each analysis.

The VSM analysis was performed using a Quantum Design PPMS DynaCool 9T system for both the nanoparticles and the solid nanocomposite disks. For nanoparticles, an aliquot of 200 μL of the colloidal nanoparticle solution was diluted in 10 mL of ethanol. Subsequently, 460 μL of this solution (then containing 8.74 × 10^–5^ g of iron oxide) was deposited onto a Teflon surface placed on a heating plate set to 90 °C. This step aimed to rapidly evaporate the ethanol, leaving behind only the IONPs adhered to the Teflon. After complete evaporation of the ethanol and drying of the particles, the Teflon was carefully rolled and inserted into the sample holder of the VSM equipment. The measurements were conducted over a temperature range of 50 to 330 K for the magnetization versus magnetic field (*M*–*H*) studies, and at 1591.54 Am^‑1^ (20 Oe) and 1 T for the magnetization versus temperature (*M*–*T*) studies. We conducted magnetization measurements as a function of temperature using the zero field cooling (ZFC) and field cooling (FC) protocols, as well as magnetization measurements as a function of the magnetic field. For the ZFC protocol, the sample was heated to the highest measurement temperature without an applied magnetic field and then cooled to 3 K. A magnetic field of 20 Oe was then applied, and the magnetization was measured while the temperature increased at a rate of 1 K min^–1^. For the FC protocol, the sample was heated to the highest measurement temperature with a field of 20 Oe applied, then cooled to 3 K, and the magnetization was measured as the temperature increased at the same rate used in the ZFC protocol. The magnetization (*M*) of a superparamagnetic nanoparticle as a function of the applied magnetic field (*H*) is described by the Langevin function:

3 $$M\left(H\right)=M_{s}\left(\coth\left(\frac{\mu H}{\mathrm{kT}}\right)-\frac{\mathrm{kT}}{\mu H}\right)$$

where *M*_s_ is the saturation magnetization, μ is the mean magnetic moment of the nanoparticles, *k* is the Boltzmann constant, and *T* is the temperature. Data analysis was performed using the Origin 2024 software, fitting the obtained magnetization curves to the Langevin model and comparing them with reference data from the literature to determine the magnetic properties and behavior of the samples.

For the nanocomposites, a piece of sample was cut and wrapped in Teflon. The sample was then mounted on a VSM sample holder. Measurements were conducted at 300 K for magnetization versus magnetic field (M–H) studies, with the applied magnetic field ranging from −30 to 30 kOe. For magnetization versus temperature (M–T) studies, measurements were taken at a fixed magnetic field of 20 Oe. Data analysis was performed using the Origin 2024 software, fitting the obtained magnetization curves to the Langevin model. Both the hyperthermia and VSM results are presented after normalization by the mass of nanoparticles in each sample determined by TGA.

The heating efficiency of the magnetic polymeric nanocomposites was evaluated via calorimetric measurements. The samples were weighed and dispersed in 2.0 mL of deionized water to ensure thermal homogeneity. The measurements were conducted using a magnetic hyperthermia system of a magneTherm from nanoTherics under an alternating magnetic field (AMF). The experiments were performed at a fixed frequency (*f*) of 174.1 kHz and a magnetic field amplitude (*B*) of 50 mT, corresponding to a magnetic field strength (*H*) of approximately 39.8 kA/m. The temperature evolution of the samples was recorded as a function of time using a fiber-optic temperature probe immersed in the dispersion. The Specific Absorption Rate (SAR) was calculated using the initial slope method, according to the following equation:

4 $$\mathrm{SAR}=\frac{C_{p}\cdot m_{\mathrm{total}}}{m_{\mathrm{NP}}}\cdot \left(\frac{dT}{dt}\right)$$

Where *C*_p_ is the specific heat capacity of the medium, assumed as the value of water, 4.184 J·g^–1^·K^–1^. The *m*_(total)_ is the total heated mass (sample mass + 2.0 g of water), and the *m*_NP_ is the mass of magnetic nanoparticles, calculated from the mass fraction determined by TGA (8.8 wt % for hydrophobic and 1.3 wt % for hydrophilic samples). The variables *T* and *t* represent the absolute temperature and time, respectively, and the fraction d*T*/d*t* corresponds to the initial slope of the temperature vs time curve, obtained by linear regression of the first 40 s of heating. To allow comparison with other systems independent of the specific field parameters, the Intrinsic Loss Power (ILP) was calculated as

5 $$\mathrm{ILP}=\frac{\mathrm{SAR}}{f\cdot H^{2}}$$

Where SAR is the Specific Absorption Rate, *f* is the fixed frequency of the alternating magnetic field, and *H* is the magnetic field strength. Data analysis was performed using Origin 2024 software to generate temperature versus time curves.

## Results

DLS analysis was performed to assess the size distribution and uniformity of the synthesized hydrophilic and hydrophobic nanoparticles (Figure 1). The data were evaluated using two distinct algorithms: the cumulant method to obtain the overall *Z*-average and polydispersity index (PDI) and the general model to extract the intensity-weighted size distributions. For the hydrophilic nanoparticles synthesized with triethylene glycol (TEG), the cumulant analysis across multiple measurements yielded overall *Z*-average diameters ranging from 5.4 to 8.4 nm, with corresponding PDI values ranging from 0.14 to 0.27. Consistently, the general model analysis for the intensity-weighted size distributions (Figure 1) revealed peak hydrodynamic sizes ranging from 5.7 to 10.7 nm. The low overall PDI and narrow intensity peaks indicate that the hydrophilic nanoparticles are highly uniform, making them well-suited for applications requiring superparamagnetic properties.

**Figure 1:**
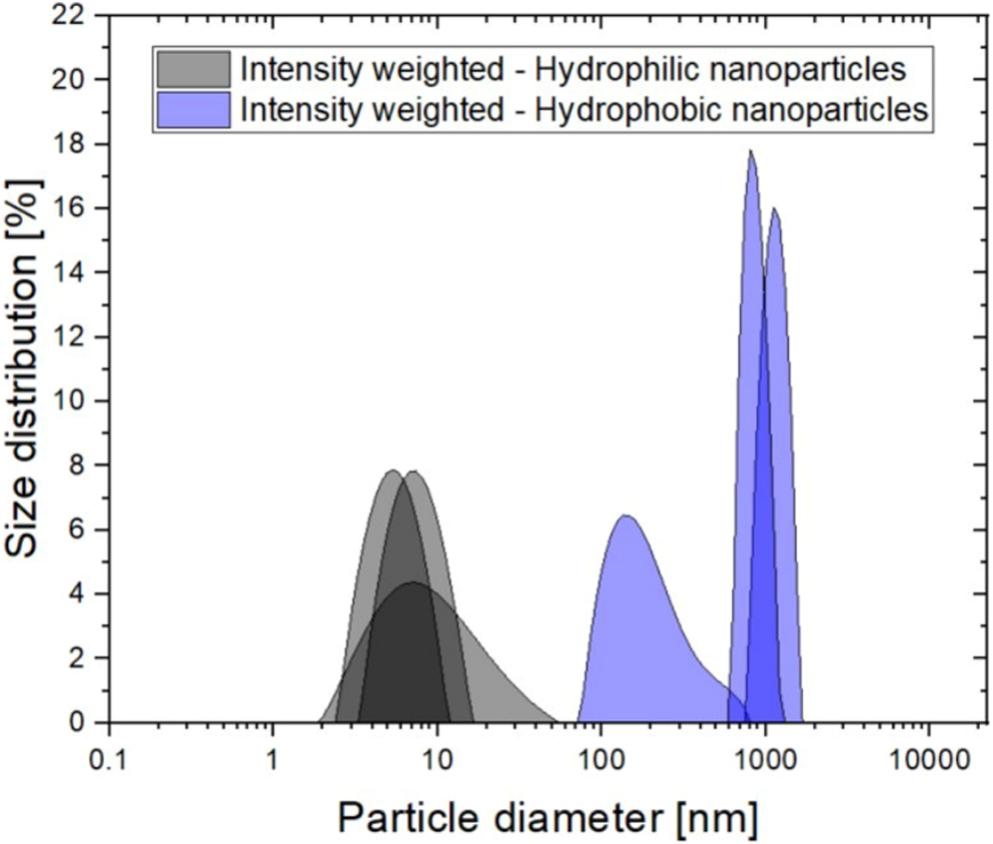
Intensity-weighted particle size distribution curves obtained from DLS measurements of hydrophilic (TEG) and hydrophobic (BA) samples. The three individual curves for each type of nanoparticle represent three independent measurements, highlighting the reproducibility of the hydrophilic nanoparticles and the massive agglomeration and high measurement-to-measurement variability of the hydrophobic nanoparticles.

In contrast, the hydrophobic nanoparticles synthesized with benzyl alcohol displayed high variability and massive agglomeration across different measurements (as shown by the three individual curves in Figure 1). According to the cumulant analysis, the overall *Z*-average diameters for the hydrophobic samples varied significantly between measurements, ranging from 266 to 1525 nm, with PDI values ranging from 0.12 to 0.28. Respectively, the general model analysis for the intensity-weighted size distributions revealed highly shifted hydrodynamic peak sizes ranging from 213 nm up to 1147 nm. The significantly larger diameters and the massive measurement-to-measurement discrepancy confirm a highly polydisperse system. This agglomeration may be attributed to the solvent used for DLS analysis, which could destabilize the hydrophobic particles, highlighting the need to consider the solvent effects in nanoparticle characterization. This discrepancy in size compared to the hydrophilic nanoparticles warrants further investigation using additional characterization techniques, such as transmission electron microscopy (TEM), to verify the accuracy of these measurements.

The average size of the nanoparticles was also measured by transmission electron microscopy (TEM). For the hydrophilic nanoparticles, TEM images show their aggregated state (Figure 2a) and partially spherical morphology (Figure 2b), while the corresponding Gaussian fit from the size distribution confirms an average size of 5.5 nm (Figure 2c). Similarly, for the hydrophobic nanoparticles, images depict their aggregation (Figure 2d) and spherical morphology (Figure 2e), with the Gaussian fit showing an average size of 9.7 nm (Figure 2f).

**Figure 2:**
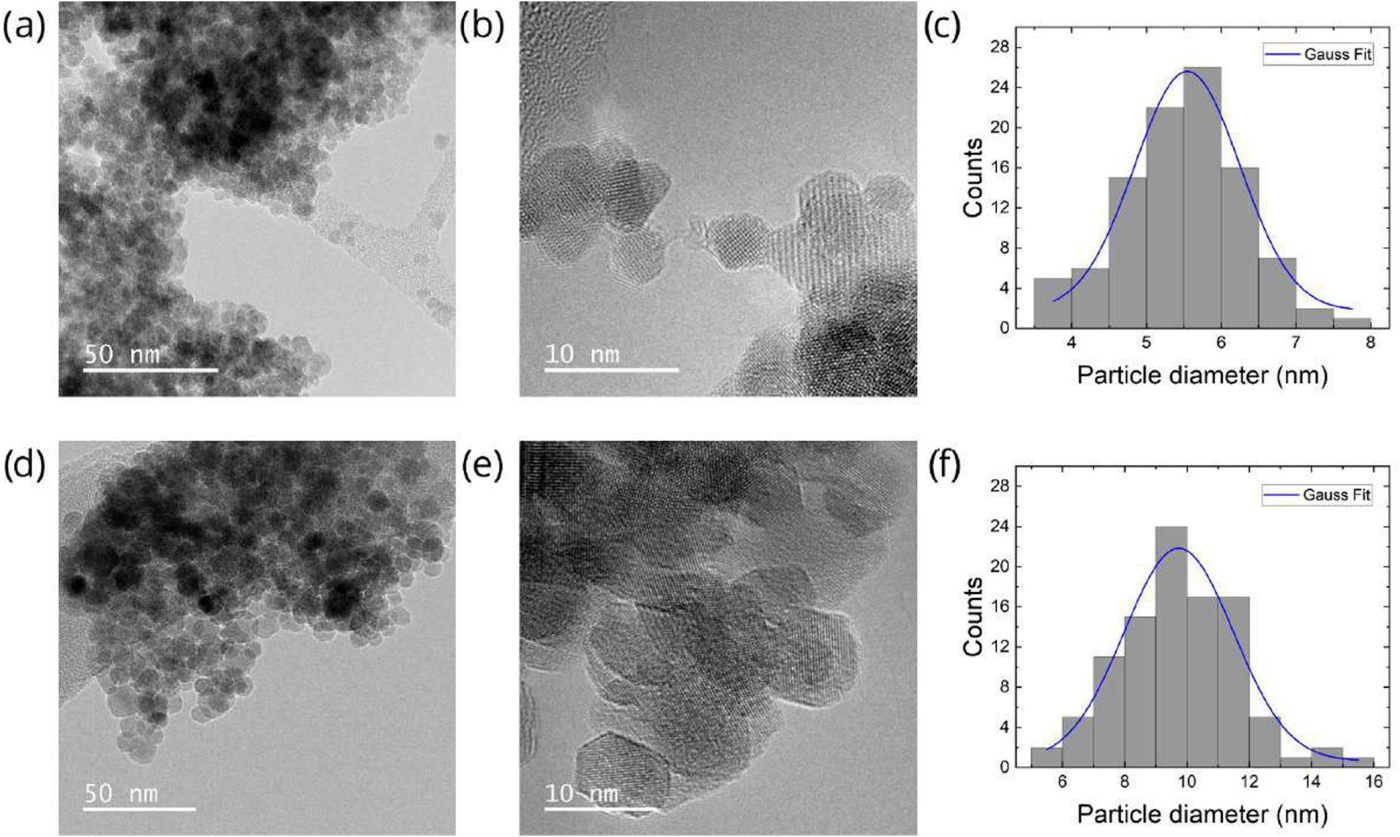
Transmission Electron Microscopy images of hydrophilic nanoparticles with a (a) 50 nm scale bar and (b) 10 nm scale bar, with (c) a histogram of hydrophilic nanoparticles. TEM image of hydrophobic nanoparticles with a (d) 50 nm scale bar and (e) 10 nm scale bar, with (f) histogram of hydrophobic nanoparticles.

The size of the hydrophilic nanoparticles was shown to be consistent across both analytical techniques employed, namely, transmission electron microscopy (TEM) and dynamic light scattering (DLS). In contrast, for the hydrophobic nanoparticles, a notable discrepancy between the two methods was observed. This is attributed to the solvent used for the DLS analysis, which promoted nanoparticle aggregation. Consequently, the DLS measurements reflected the hydrodynamic diameters of these agglomerates and not the primary sizes of the individual nanoparticles.

To accurately quantify the actual nanoparticle loading, which is central for the subsequent normalization of the magnetization and hyperthermia data (SAR/ILP), Thermogravimetric Analysis (TGA) was performed. As detailed by the representative TGA curves now included as an inset in Figure 9 (with full profiles in the Supporting Information, Figures S1–S2 and Tables S1–S2), the thermal decomposition of the nanocomposites presents distinct mass loss stages. Following the initial evaporation of water and residual solvents, the major mass loss occurs between approximately 160 and 460 °C, corresponding to the degradation of the polymer matrix and the decomposition of the organic surface ligands (TEG or BA). After the complete thermal degradation of the organic components and unhydrolyzed groups, the final stable residual mass yielded the actual inorganic iron oxide core fractions: 1.3 wt % for the nanocomposite loaded with hydrophilic IONPs, and 8.8 wt % for the nanocomposite loaded with hydrophobic IONPs. These precise mass fractions were intrinsically used to normalize the performance metrics in the magnetic and calorimetric evaluations.

Figure 3 shows the XRD patterns of the neat Siloxane-PEO-PPO hybrid and of the same hybrid loaded with 15.1% and 17.8 wt % of hydrophilic and hydrophobic iron oxide particles, respectively. Note that these mass fractions correspond to the proportion of iron oxide regarding the polymer phase and not the whole samples, since they were determined from TGA measurements by subtracting the contribution of remaining water and ethanol resulting in incomplete drying of the samples and of the organic ligands located at the particle’s surface (see Figures S1 and S2 of Supporting Information and the corresponding Tables S1 and S2). The peaks positions of the iron oxide crystalline phases of maghemite and magnetite are also presented in Figure 3.

**Figure 3:**
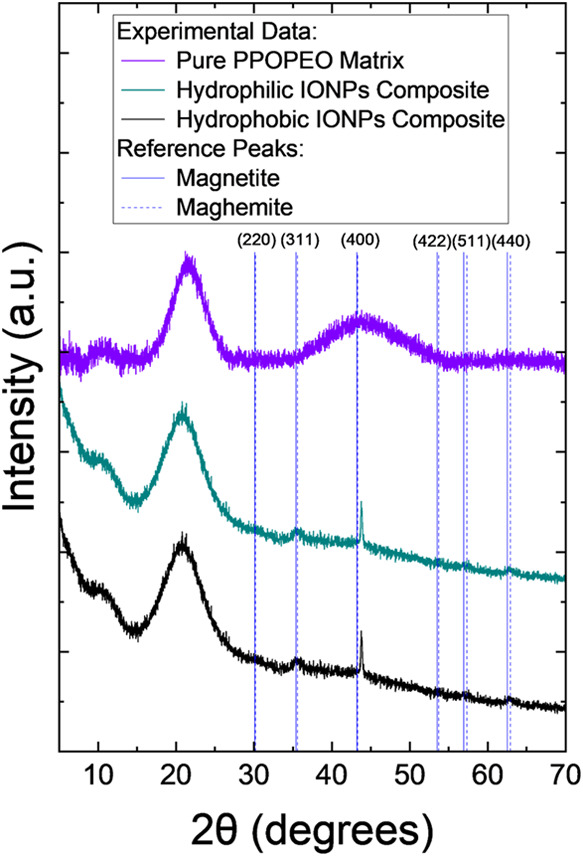
X-ray diffraction (XRD) patterns of the pure siloxane-PPO-PEO (70–30) matrix, and the nanocomposites siloxane-PPO-PEO-IONPs (70–30) loaded with either hydrophilic or hydrophobic iron oxide nanoparticles. The reference peak positions for the crystalline phases of magnetite and maghemite are also presented to allow proper identification.

The pure hybrid matrix displays the well-known characteristic broad bands centered around 2θ = 21.7 and 43° related to the amorphous siloxane and polymer phases, respectively. Interestingly, a relatively broad peak centered around 2θ = 10.8° appears in the pattern. Such a type of peak has already been observed in Siloxane-Polymer hybrids containing urea or urethane groups and attributed to a periodicity between the organic chains promoted by hydrogen bonds formed between the urea or urethane moieties located at chains extremities.^40^ In the present study the average interchain distance corresponds to 0.82 nm.

Note that this peak is still present around 2θ = 11.1° and 2θ = 11.4° in the patterns of the solid nanocomposite disks loaded with hydrophilic and hydrophobic nanoparticles, respectively. Furthermore, the expected crystalline phases of iron oxide (magnetite and/or maghemite) are present in the siloxane-PPO-PEO matrix. This is confirmed by the distinct diffraction peaks observed in the nanocomposite patterns at 2θ values of approximately 30.2, 35.6, 43.3, 53.7, 57.3, and 62.9°, which correspond respectively to the (220), (311), (400), (422), (511), and (440) crystallographic planes of the inverse spinel structure.^41,42^ These peaks exhibit lower intensity and slight peak shifts compared with the patterns of the isolated nanoparticles (see Figure S3 of Supporting Information). Another interesting result is that the broad band related to the amorphous polymer phase disappears in the patterns of both loaded nanocomposites.

The FTIR spectrum of the neat siloxane-PPO-PEO hybrid (Figure 4) displays several characteristic bands. The fact that the broad band is centered around 3356 cm^–1^ reveals the existence of N–H stretching vibrations of hydrogen bonded urea groups.^20^ When N–H groups of urea or urethane are hydrogen bonded, a band located between 3300 and 3400 cm^–1^ is detected in the FTIR spectra, while a signal associated with free N–H groups appears at frequencies larger than 3400 cm^–1^.^20^ Such interaction between urea groups agrees with the presence of the ordered “lamellar” structure between polymer chains suggested by XRD measurements. Note that this signal is convoluted with the well-known broad band usually located between 3200 and 3700 cm^–1^ attributed to stretching of hydroxyls groups of water molecules, which have not been removed during gel drying. Another bands related to urea groups are located at 1639 and 1562 cm^–1^. The former is known as amide I and is attributed to a mixed contribution of C = O stretching, C–N stretching and C–C–N deformation.^20^ The latter is the amide II mode, a mixed contribution of the N–H in-plane bending, the C–N stretching, and the C–C stretching vibrations.^20^ Both bands are sensitive to chain conformation and intermolecular hydrogen bonding. The bands related to the polymer phase are located at 2869 cm^–1^ (attributed to C–H_2_ stretching), at 1450 cm^–1^ (associated with CH, scissoring and CH_3_ deformation), at 1376 and 1253 cm^–1^ (CH_2_ wagging and twisting, respectively), and 880 cm^–1^ (C–O stretching and CH_2_ rocking vibrations).^20^ Note that due to the high polymer content in the material (see Figure S2 and Table S1 of Supporting Information), the vibrations corresponding to the siloxane phase (Si–O–Si) and silicon atom (C–Si–O) are masked by the strong band split at 1085 and 1049 cm^–1^, which corresponds to the C–O stretching of PEO and PPO.^20^ Finally, the small band around 2972 cm^–1^ corresponds to asymmetric CH_3_ stretching and originates from the nonhydrolyzed ethoxy groups of the hybrid precursors, revealing that sol–gel reactions (hydrolysis and condensation of the silicon species) are not totally completed.

**Figure 4:**
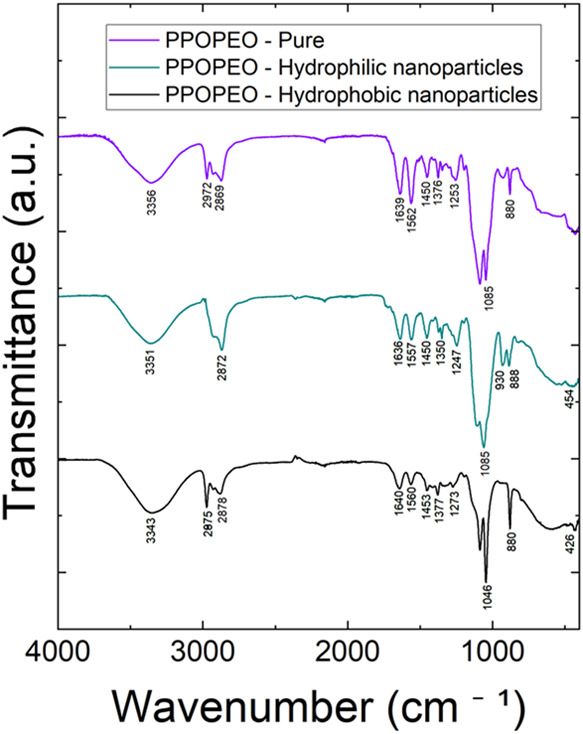
FTIR spectra, from top to bottom, of pure siloxane-PPO-PEO (70–30) matrix and of the nanocomposites siloxane-PPO-PEO-IONPs (70–30) with either hydrophilic or hydrophobic iron oxide nanoparticles.

In the spectrum of the solid nanocomposite disks loaded with hydrophilic nanoparticles, we noticed the shift of the amide I and amide II bands to 1636 and 1557 cm^–1^, respectively, and the pronounced decrease of their intensity. Moreover, the bands related to the polymer phase show a decrease in their intensity, and some of them exhibit some shift by incorporating the hydrophilic particles (C–H_2_ stretching appears at 2872 cm^–1^, CH_2_ wagging at 1350 cm^–1^, CH_2_ twisting at 1247 cm^–1^ and C–O stretching, and CH_2_ rocking vibrations appear at 888 cm^–1^). An additional band, which was only incipient in the spectrum of the neat hybrid appears at 930 cm^–1^ in the loaded nanocomposite, corresponding to C–C stretching and CH_2_ rocking. Finally, the shape and intensity of the strong splitted band related to ether groups are clearly affected. The band at 454 cm^–1^ indicates the presence of Fe–O bonds.

The spectrum of the solid nanocomposite disks loaded with hydrophobic nanoparticles exhibits the same trends. The band related to urea–urea interactions is centered around 3343 cm^–1^, with a pronounced decrease of the intensity of the amide I and amide II bands. The decrease of intensity and some shifts of several bands related to the polymer phase are observed (C–H_2_ stretching at 2878 cm^–1^ CH_2_ scissoring and CH_3_ deformation at 1453 cm^–1^, CH_2_ wagging at 1377 cm^–1^ and CH_2_ twisting at 1273 cm^–1^), whereas the shape and intensity of the strong splitted band related to ether groups also suffer changes. The Fe–O stretching vibration is observed at 426 cm^–1^.

SEM analysis of the siloxane-PPO-PEO-IONPs (70–30) nanocomposite loaded with hydrophilic particles reveals a well-distributed dispersion of nanoparticles throughout the matrix, with no apparent preference for surface regions. The nanoparticles are predominantly aggregated into micrometer-sized clusters (Figure 5a). At higher magnifications, the detailed structure of these agglomerates can be more clearly observed (Figure 5b).

**Figure 5:**
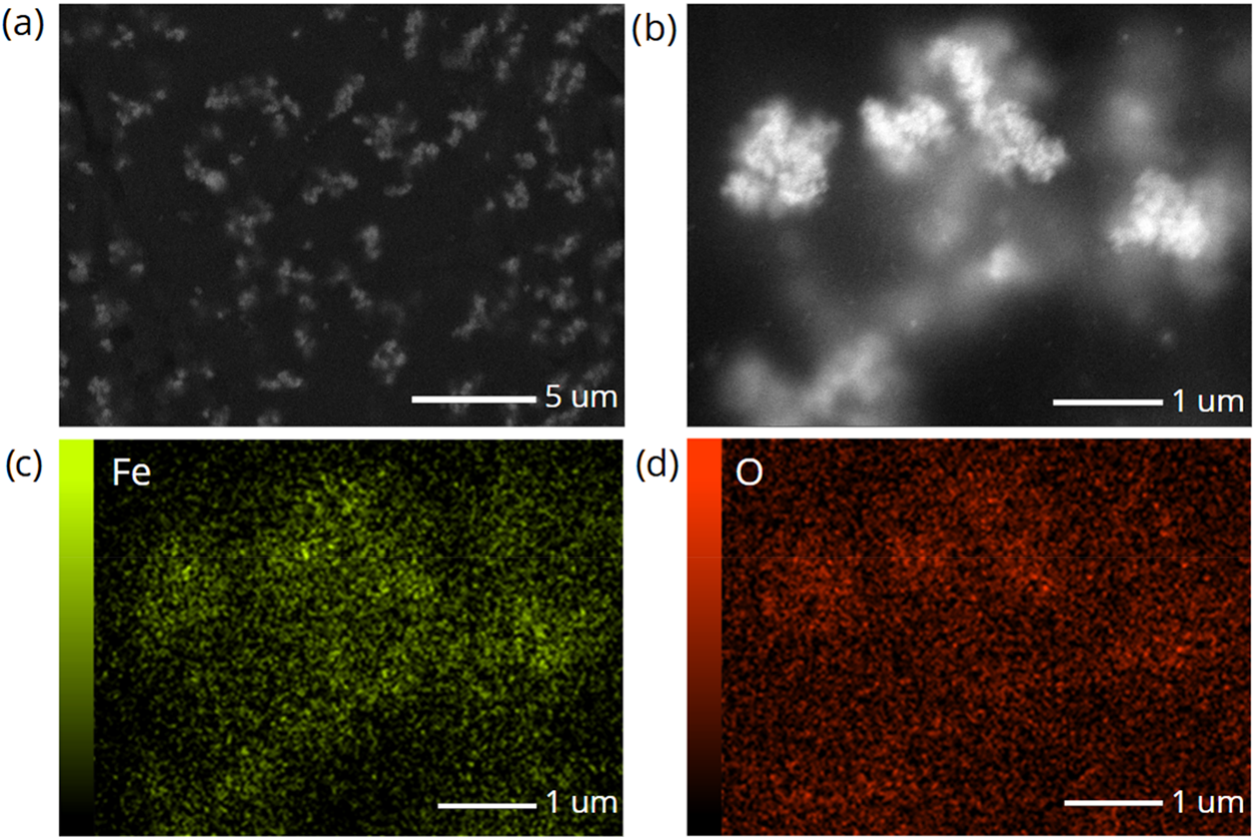
(a) SEM images of the siloxane-PPO-PEO-IONPs (70–30) nanocomposite loaded with hydrophilic particles using BED-C at 10 kV and PC 20 at ×5000 magnification, (b) EDS analysis of the siloxane-PPO-PEO-IONPs nanocomposite loaded with hydrophilic particles backscattered electron image, and (c) EDS elemental distribution map of Fe and (d) O.

To confirm that the observed structures correspond to iron oxide-rich aggregates, EDS analysis was performed on the sample. An image at 25000 magnification, 15 kV, and PC 40 was used for the EDS (Figure 5b). Elements of the nanoparticle, including oxygen and iron, were detected (Figure 5c and d). For the iron oxide, the iron spectrum appeared to be much more concentrated in the lighter structures observed, along with a higher concentration of oxygen, which is consistent with the presence of iron oxide.

Similarly, SEM analysis of the siloxane-PPO-PEO-IONPs (70–30) nanocomposite loaded with hydrophobic particles shows well-distributed micrometer-sized clusters throughout the matrix with a more spherical morphology (Figure 6a). At higher magnifications, these agglomerates can be seen with more details (Figure 6b).

**Figure 6:**
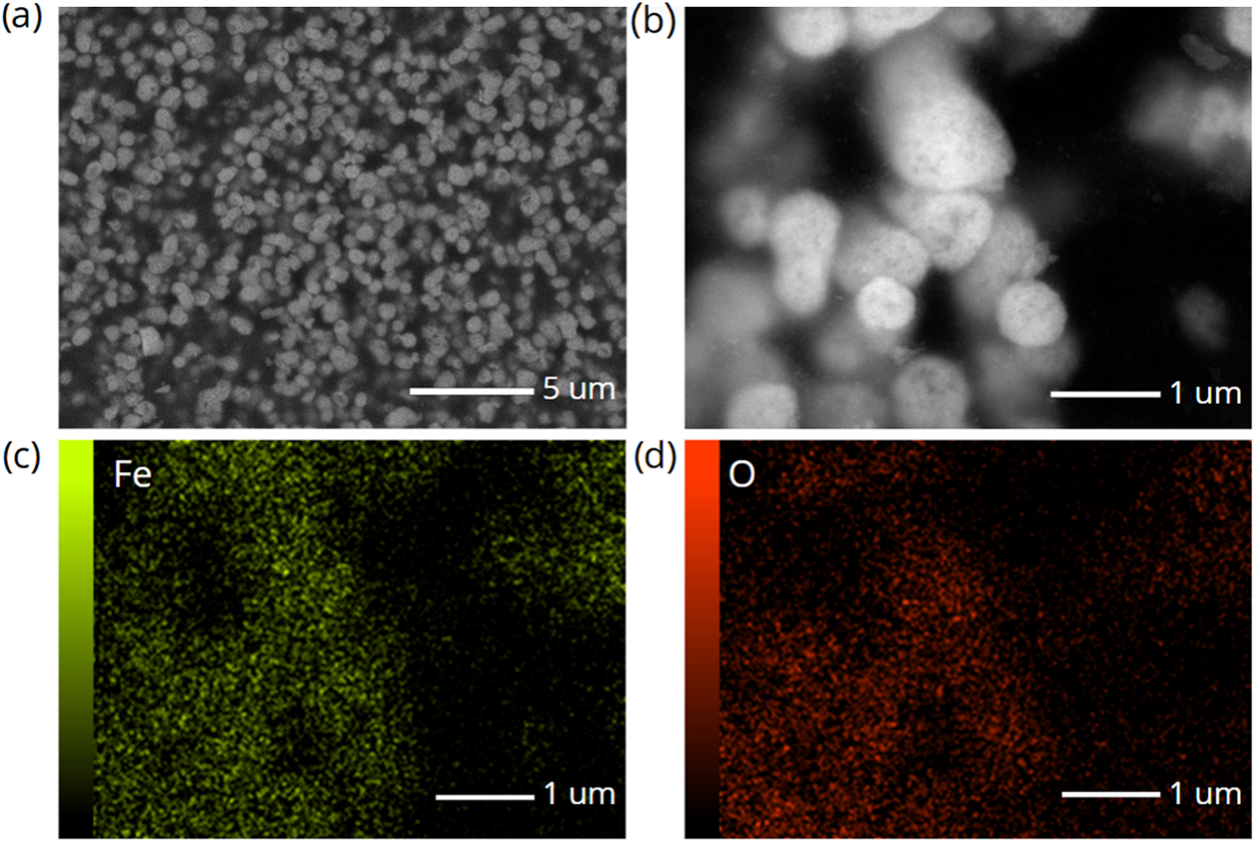
(a) SEM images of the siloxane-PPO-PEO-IONPs (70–30) nanocomposite loaded with hydrophobic particles using BED-C at 10 kV and PC 20 at x5000 magnification, (b) EDS analysis of the siloxane-PPO-PEO-IONPs nanocomposite loaded with hydrophobic particles backscattered electron image and (c) EDS elemental distribution map of Fe and (d) O.

EDS analysis was also performed on the latter sample, as shown in Figure 6c and d. Elements of the nanoparticle, including oxygen and iron, were detected. Again, for the iron oxide, the spectrum appeared much more concentrated in the lighter structures previously observed (Figure 6c), along with a higher concentration of oxygen (Figure 6d), which is consistent with the presence of iron oxide.

Figure 7 shows the SAXS curves in the log–log plot of the siloxane-PPO-PEO-IONPs (70–30) composite loaded with hydrophilic or hydrophobic nanoparticles. The SAXS pattern of the unloaded siloxane-PPO-PEO hybrid is also presented. For all samples an interference peak at higher *q*-regime is observed. Such type of peak has already been observed in SAXS patterns of pure Siloxane-PEO,^43^ Siloxane-PPO^43^ and Siloxane-PEO-PPO^29^ hybrids prepared by a similar chemical route and has also been detected in Siloxane-PPO hybrids loaded with cobalt ferrite (CoFe_2_O_4_) superparamagnetic nanoparticles.^27^ In all cases, it was attributed to the existence of a spatial correlation between the siloxane nodes connecting the PEO or PPO chains.

**Figure 7:**
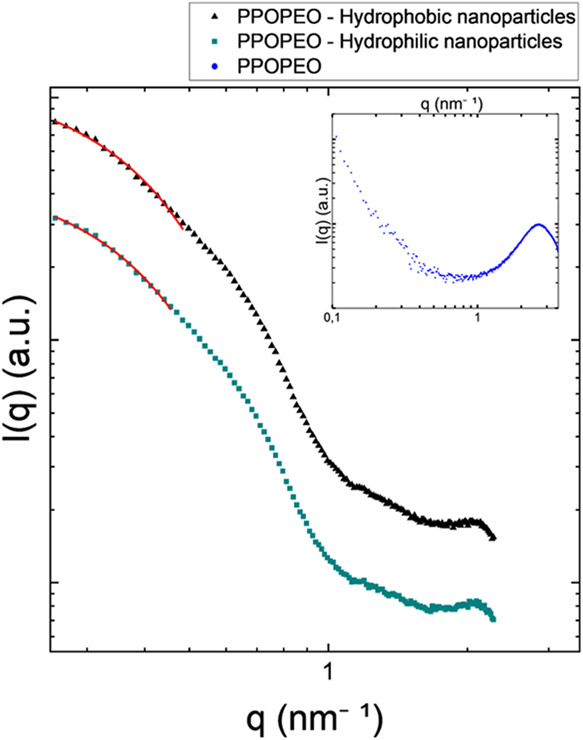
Experimental SAXS curves in a log–log plot corresponding to siloxane-PPO-PEO-IONPs (70–30) loaded with hydrophilic or hydrophobic iron oxide nanoparticles. The SAXS pattern of the pure Siloxane-PPO-PEO sample is also presented in the log–log plot in the inset.

Furthermore, a tendency to Guinier plateau at low q-values is only observed for the samples loaded with the iron oxide nanoparticles, evidencing that this X-ray scattering regime is due to an electron density contrast between iron-rich species and the solid nanocomposite disk matrix. Since the aggregates observed by SEM are of micrometer size and the SAXS technique is only sensitive to nanometer-sized objects, this regime is due to the scattering of iron oxide nanoparticles located outside the aggregates. Note that the hypothesis of a scattering regime originating from the surface of the microsized aggregates (well-known as Porod regime) can be discarded, since in this case a linear behavior in log–log plot with a slope of −4 (for smooth surface) or between −3 and −4 (for rough surface) should be observed.^44^

Figure 7 shows that the experimental SAXS curves are well-fitted at low *q*-range by the theoretical function given by eq 1. The values of the nanoparticle average radius of gyration are presented in Table 1.

**Table 1:** Estimation of the Average Radius of Gyration of the Iron Oxide Nanoparticles *R*_g_ by Using the Least-Square Method for the Fit of SAXS Curves at Low *q*-Range by the Guinier Equation^a^

Type of nanoparticle	*R*_g_ (nm)	I(0) (a.u)
Hydrophilic	4.3 ± 0.03	4844 ± 53
Hydrophobic	4.2 ± 0.04	11850 ± 152

a The results are shown for the siloxane-PPO-PEO nanocomposites loaded with hydrophilic and hydrophobic nanoparticles, along with the associated errors (obtained by the fitting procedure).

The magnetic properties of the siloxane-PPO-PEO-IONPs nanocomposites containing hydrophilic and hydrophobic IONPs, as well as those of the nanoparticles alone, were measured and are presented in Table 2. Additional data on the nanoparticles can be found in the Supporting Information (Figure S4).

**Table 2:** Magnetic Characterization of Siloxane-PPO-PEO Nanocomposites and Iron Oxide Nanoparticles

Material	Blocking Temperature (*T*_b_) (K)	Irreversibility Temperature (*T*_irr_) (K)	Saturation Magnetization (emu/g)	Average Magnetic Moment (10^–19^ emu)
Hydrophilic IONPs	66	71	76	2.60
Siloxane-PPO-PEO with Hydrophilic IONPs	94	105	58.6	4.28
Hydrophobic IONPs	236	322	47	4.80
Siloxane-PPO-PEO with Hydrophobic IONPs	103	264	11.8	4.48

The VSM analysis of siloxane-PPO-PEO-IONPs nanocomposites with hydrophilic and hydrophobic nanoparticles revealed differences in their magnetic properties (Figure 8). The blocking temperature (*T*_b_) for the composites loaded with hydrophilic IONPs is observed at 94 K, and the irreversibility temperature (*T*_irr_) at 105 K. The saturation magnetization (*m*_0_) of this composite is 58.6 emu g^–1^, with an average magnetic moment (μ) of 4.28 × 10^–19^ emu.

**Figure 8:**
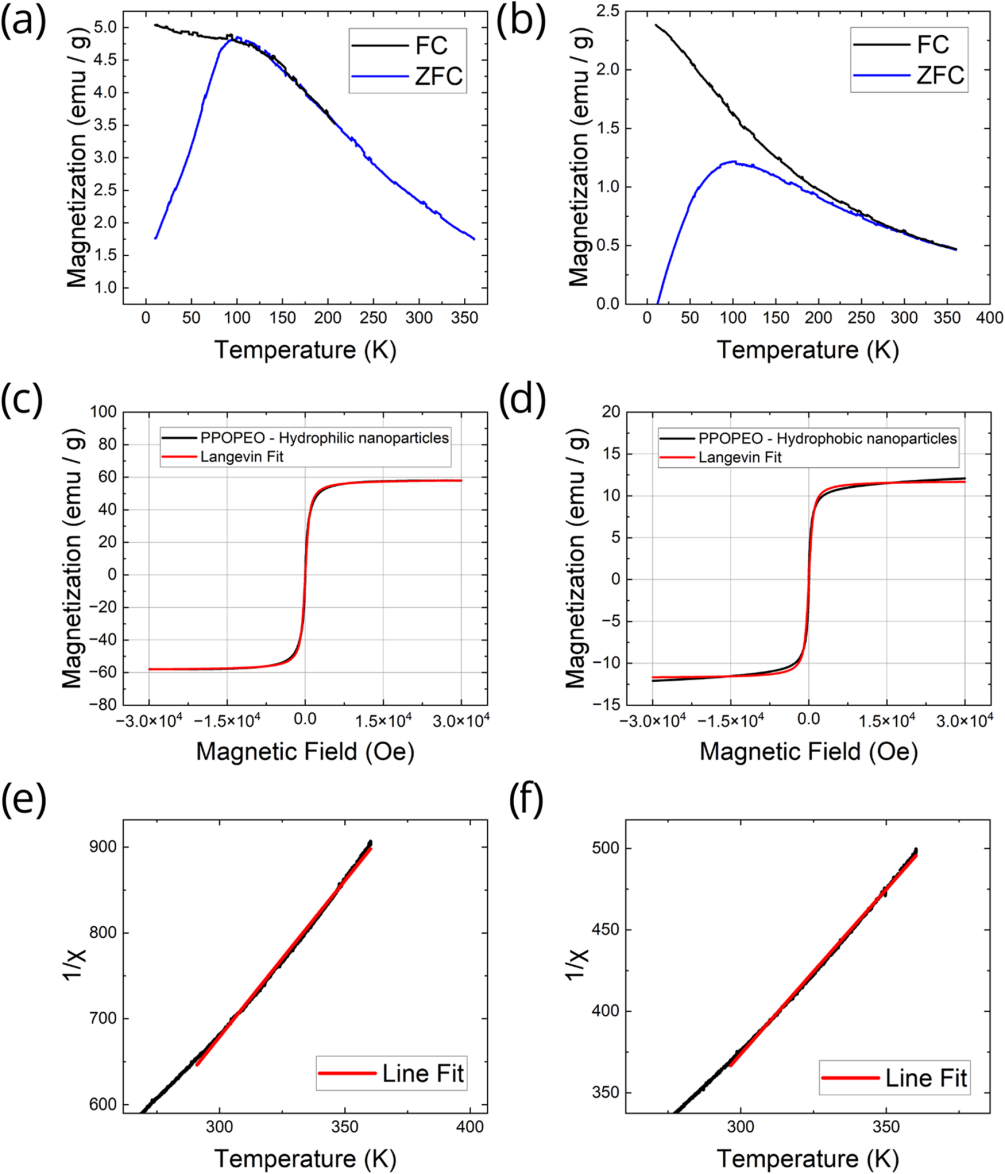
Magnetic characterization of Siloxane-PPO-PEO nanocomposites loaded with either hydrophilic or hydrophobic iron oxide nanoparticles. (a) ZFC and FC magnetization curves for solid nanocomposite disks loaded with hydrophilic nanoparticles as a function of temperature, showing a blocking temperature (*T*_b_) of 94 K and an irreversibility temperature (*T*_irr_) of 105 K. (b) ZFC and FC magnetization curves for solid nanocomposite disks loaded with hydrophobic nanoparticles, showing *T*_b_ at 103 K and *T*_irr_ at 264 K. (c) Magnetization curve at 300 K fitted with the Langevin function, resulting in a saturation magnetization of 58.6 emu g^–1^ and an average magnetic moment of 4.28 × 10^–19^ emu. (d) Magnetization curve at 300 K fitted with the Langevin function, resulting in a saturation magnetization of 11.8 emu g^–1^ and an average magnetic moment of 4.48 × 10^–19^ emu. (e) Inverse magnetic susceptibility (1/χ) versus temperature for solid nanocomposite disks loaded with hydrophilic nanoparticles, indicating linear behavior above 300 K. (f) Inverse magnetic susceptibility (1/χ) versus temperature for solid nanocomposite disks loaded with hydrophobic nanoparticles, showing linear behavior above 300 K.

In contrast, for composites loaded with hydrophobic nanoparticles, the *T*_b_ is observed at 103 K, and the *T*_irr_ at 264 K, values significantly lower than those measured for the hydrophobic nanoparticles (Figure S4 of Supporting Information). In this case, there is a larger difference between *T*_b_ and *T*_irr_ and a significantly lower saturation magnetization for this composite (11.8 emu g^–1^, with an average magnetic moment of 4.48 × 10^–19^ emu) compared to hydrophobic nanoparticles. In both types of composites, the field-dependent magnetization (M vs H) curve is not perfectly fitted by the Langevin function. However, this behavior is more pronounced for solid nanocomposite disks loaded with hydrophobic particles.

To verify their superparamagnetic behavior, the curve *χT* was also plotted (Figure 8e and f), this overlap yields a linear response above 291 K for the hydrophilic nanoparticles and 297 K for the hydrophobic nanoparticles.

The calorimetric measurements of the siloxane-PPO-PEO magnetic nanocomposites were performed under an alternating magnetic field of 50 mT and 174.1 kHz. The samples were dispersed in 2.0 mL of water, and the temperature increase was recorded over time. The specific absorption rate (SAR) and intrinsic loss power (ILP) values were calculated based on the maximum heating rate (slope) observed during the exposure. The obtained parameters are summarized in Table 3.

**Table 3:** Magnetic Hyperthermia Parameters for the Analyzed Samples (*f* = 174.1 kHz, *B* = 50 mT)

Sample	Sample Mass (mg)	NP Fraction (wt %)	Max Slope (K/s)	SAR (W·g^–1^)	ILP (nH·m^2^·kg^–1^)
Hydrophilic 1	33.05	1.3	0.0073	145.31	0.53
Hydrophilic 2	33.03	1.3	0.0070	139.00	0.50
Hydrophilic 3	38.63	1.3	0.0054	91.76	0.33
Hydrophobic 1	12.77	8.8	0.0064	48.04	0.17
Hydrophobic 2	16.49	8.8	0.0060	35.10	0.13
Hydrophobic 3	11.98	8.8	0.0033	26.73	0.10

For a better visualization and direct comparison of the heating efficiency across the different systems, the SAR and ILP data are presented in Figure 9.

**Figure 9:**
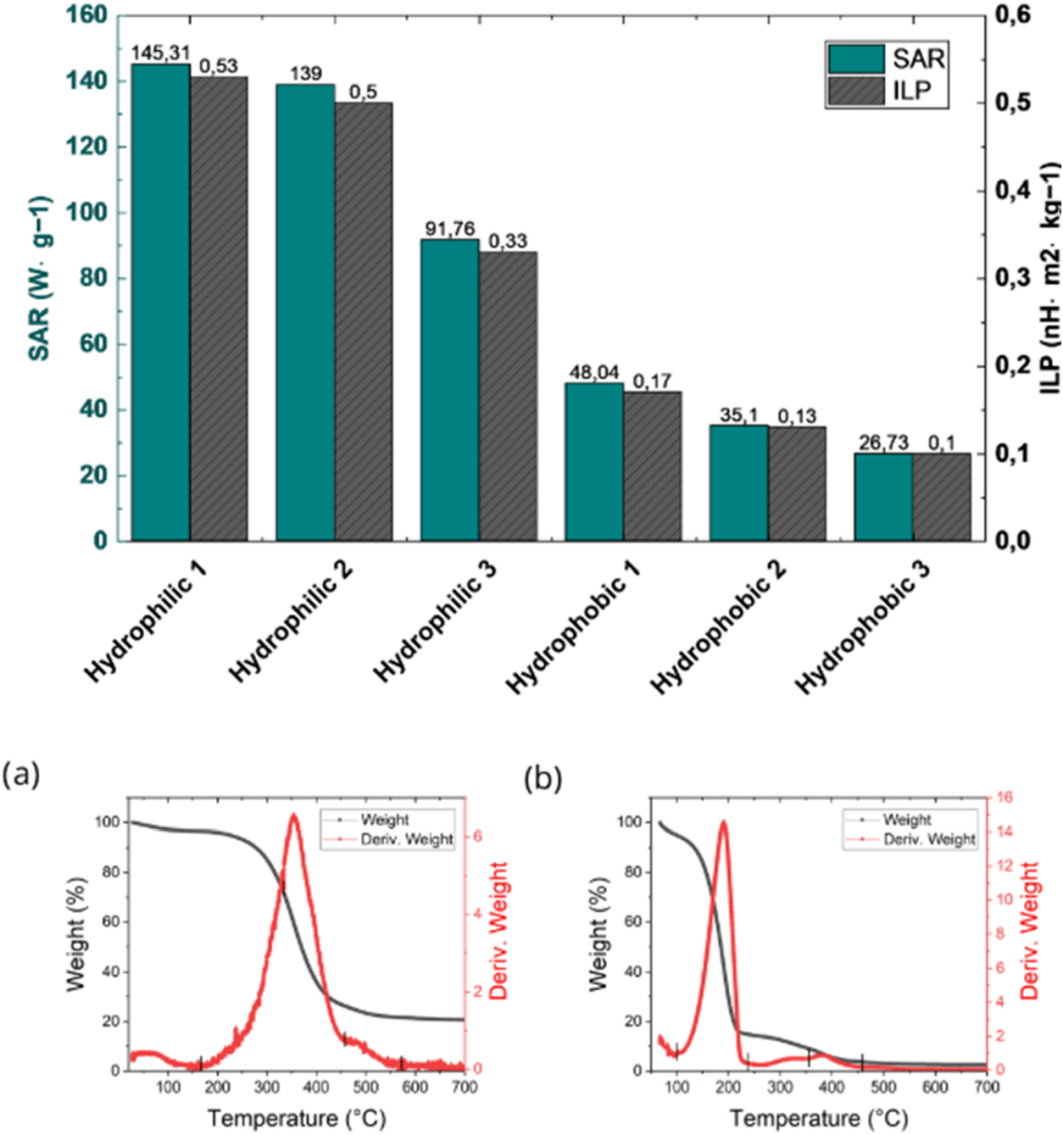
Comparison of magnetic hyperthermia performance parameters (SAR and ILP) for hydrophobic and hydrophilic samples with the representative thermogravimetric analysis (TGA) curves used to determine the inorganic mass fraction for SAR normalization of (a) siloxane-PPO-PEO-IONPs nanocomposite with hydrophobic nanoparticles and (b) siloxane-PPO-PEO-IONPs nanocomposite with hydrophilic nanoparticles.

The samples loaded with hydrophilic nanoparticles (Hydrophilic 1, 2, and 3) exhibited immediate temperature increments upon field application. The maximum heating rates for this group ranged from 0.0054 to 0.0073 K/s. Consequently, the calculated SAR values were between 70.8 and 145.3 W/g, while the ILP values ranged from 0.26 to 0.53 nH·m^2^/kg.

The solid nanocomposite disks loaded with hydrophobic nanoparticles (Hydrophobic 1, 2, and 3) showed lower heating efficiency under the same experimental conditions. The SAR values for this group varied from 26.7 to 48.0 W/g, corresponding to ILP values between 0.10 and 0.17 nH·m^2^/kg. Specifically regarding the Hydrophobic 3 sample, the maximum slope was not observed in the initial instants of measurement but was detected in the time interval between 160 and 200 s.

Comparing the two groups, the hydrophilic samples presented SAR and ILP values approximately 3 to 4 times higher than those obtained for the hydrophobic samples, despite the lower mass fraction of magnetic nanoparticles (1.3 vs 8.8 wt %).

## Discussion

In the XRD patterns, the peak attributed to the periodicity between the organic chains indicates that IONP incorporation does not disrupt this organization. Furthermore, the presence of the expected iron oxide crystalline phases confirms the successful embedding of IONPs within hybrid matrices. The disappearance of a broad band related to the amorphous polymer phase in the patterns of loaded nanocomposites indicates that nanoparticles are located in the free volume of the polymer phase. Such good interaction with the polymer chains for both types of IONPs (hydrophilic or hydrophobic) should be due to the presence in the hybrid matrix of both hydrophilic PEO chains and hydrophobic PPO chains, improving the dispersion of each type of particles in the polymer phase. This is crucial for the intended applications, since a high chemical affinity between the polymer and the IONPs should favor a uniform particle dispersion in the matrix, enhancing the mechanical, thermal, and magnetic properties of the nanocomposites.

The pronounced decrease of intensity and shift of the amide I and amide II bands in the FTIR spectrum of the solid nanocomposite disks loaded with hydrophilic particles reveal the existence of interactions between some urea groups and some nanoparticles, possibly facilitated by hydrogen bonding with organic compounds located at the nanoparticle surface. However, incorporation of iron oxide particles does not inhibit formation of hydrogen bonds between other urea groups, attested by the presence of the band centered at 3351 cm^–1^. Again, this result agrees with the presence of the peak around 2θ = 11° in the XRD pattern of this sample (ordering between polymer chains promoted by urea–urea interactions). The observed changes in intensity and position of bands related to the polymer phase confirm that IONPs also interact with the polymer matrix, already suggested by XRD measurements. The spectra of the solid nanocomposite disks loaded with hydrophobic nanoparticles are similar and also corroborates XRD results. The decrease in the intensity of the amide I and amide II bands evidences the interactions between urea moieties and some nanoparticles. The iron oxide nanoparticles-polymer interactions are revealed by the decrease and shifts of the bands related to the polymer phase.

Specific attention was paid to the potential formation of covalent bonds between the IONPs and the siloxane nodes of the hybrid network. The formation of Si–O–Fe linkages through condensation reactions between silanol groups of the hydrolyzed hybrid precursor and hydroxyl groups located at iron oxide particles surface would typically yield a characteristic absorption band in the 450–569 cm^–1^ range. However, the spectrum of the nanocomposite containing hydrophobic nanoparticles displays no distinct absorption features in this region. The observed band at approximately 426 cm^–1^ is attributed solely to the intrinsic Fe–O lattice vibration of the iron oxide core. The absence of the Si–O–Fe signature clearly evidence that the iron oxide nanoparticles are not chemically grafted to the siloxane phase. Consequently, the IONPs are likely dispersed within the matrix primarily through noncovalent interactions with the organic segments, specifically the favorable acid–base interactions between the iron oxide surface and the ether groups of the PPO/PEO chains, rather than through direct chemical modification by the inorganic cross-links.

SEM and EDS also show similarities between hydrophilic and hydrophobic nanoparticles when they are incorporated in the hybrid matrix. These techniques evidence in both cases the presence of iron oxide aggregates of micrometer size exhibiting a uniform distribution and a relatively low size dispersion, which is crucial for maintaining the desired properties of the nanocomposites. However, the different shape of the iron oxide clusters between the solid nanocomposite disks loaded with hydrophilic particles and the solid nanocomposite disks loaded with hydrophobic ones suggests a different interaction mechanism with the matrix. Furthermore, the observed aggregation of IONPs in microsized domains detected by SEM does not corroborate the XRD and FTIR results, which evidence significant interactions between IONPs and different coordination sites of the hybrid matrix. This justified the investigation of the composite nanostructure by SAXS.

The presence of a Guinier regime attributed to IONPs scattering in the SAXS patterns of the loaded solid nanocomposite disks should at first be surprising, since it is expected in “diluted” systems containing low concentrations of nanosized scatterers in the matrix.^38^ However, SEM results reveal a high content of IONPs in the matrixes at the micrometer scale, while the SAXS technique investigates the material’s structure at the nanometer scale. We then conclude that the regions located between the aggregates detected by SEM form a diluted system of well-dispersed IONPs in the matrix at nanometer scale. This result agrees with XRD and FTIR measurements, which have evidenced the existence of particle–matrix interactions, favoring iron oxide particle dispersion in the matrix. Despite the low extension of the plateau resulting in the limitations of our experimental setup, an estimation of the average radius of gyration *R*_g_ of the nanoparticles can be determined by using the Guinier equation (eq 1),^38^ and the values are of the same order as the iron oxide nanoparticles radius values determined by TEM.

SAXS curves also revealed that the intensity of the peak related to spatial correlation between the siloxane nodes is significantly reduced in IONPs-loaded samples comparing to the IONPs-free hybrid, due to the higher electron density contrast between the IONPs and the polymer matrix as compared to the electron density contrast between the siloxane nodes and the polymer, which contributes to “mask” the signal originating from the siloxane phase. This interpretation has already been proposed for Siloxane-PPO hybrids loaded with cobalt ferrite (CoFe_2_O_4_) superparamagnetic nanoparticles, which exhibited the same behavior.^27^ The presence of this peak confirms the successful formation of the siloxane nodes as cross-links between polymer chains and the formation of a tridimensional hybrid network, which is consistent with the solid monoliths obtained in this work. The average distance d between the siloxane nodes can be estimated from the peak position through eq 2. As expected, the *d* value (3 nm) is similar for both loaded samples since the weight fraction of PEO and PPO is the same in both samples (the average chain length of PEO being greater than the PPO one in this study, as already specified). Note that a contribution of the IONPs located outside the microsized aggregates to the interference peak can be discarded, since in this case a shift of the peak position toward higher q-value (corresponding to a decrease of the distance between all nano-objects) by loading the hybrid material with IONPs should occur. On the contrary, a shift of the peak toward lower q-value is observed by comparing the pattern of the loaded samples with the IONPs-free one, revealing an increase of the distance between siloxane nodes from 2.4 to 3 nm. This may be attributed to slight changes in polymer chain conformation due to their interactions with IONPs. Furthermore, the fact that the IONPs do not chemically interact with the siloxane nodes, as revealed by FTIR results, also confirms that the SAXS peak only originates from a spatial correlation between the siloxane nanodomains.

Differences between interactions of hydrophilic and hydrophobic particles with the hybrid matrix are more apparent when their magnetic properties are investigated. In the VSM analysis, the proximity between *T*_b_ and *T*_irr_ values in the composites loaded with hydrophilic IONPs indicates weak interactions between nanoparticles. However, these values are higher than those measured for the IONPs (Figure S4 of the Supporting Information), which may be related to a slight aggregation of the hydrophilic nanoparticles when incorporated into a predominantly hydrophobic matrix. The decrease in saturation magnetization for the same composite means that the nanoparticles have lost their magnetic moment. This can be attributed to a loss of coating when inserted into the polymer matrix, thus generating a disorder of the surface magnetic moments. As for the increase in the average magnetic moment, this is closely related to the increase in the blocking temperature and, therefore, is attributed to the slight aggregation of the nanoparticles.

For the composites loaded with hydrophobic nanoparticles, the lower *T*_b_ and *T*_irr_ values indicate that incorporation in the matrix reduces the magnetic moment and thermal stability of the superparamagnetic state. The larger difference between *T*_b_ and *T*_irr_ and the significantly lower saturation magnetization (*m*_0_) can be plausibly explained by the strong interaction of the nanoparticles with the polymer matrix, which may partially disrupt the surface coverage, leading to increased surface disorder and a consequent reduction in the effective magnetic size of the nanoparticles. This, in turn, lowers the saturation magnetization and blocking temperature. As a result, the average magnetic moment of the nanoparticles decreases, reducing dipolar interactions, and thereby lowering the irreversibility temperature. The larger discrepancy between the experimental curve and the theoretical Langevin model observed for the solid nanocomposite disks loaded with hydrophobic particles suggests that the agglomeration of IONPs is more pronounced in this composite.

This result may be attributed to two main factors: (i) the presence of a significantly lower amount of hydrophobic ligand, as revealed by TGA measurements (Figure S2 of Supporting Information), and (ii) the disruption of surface functionalization of nanoparticles, due to stronger chemical affinity between hydrophobic particles and this essentially hydrophobic matrix. Although the higher inorganic mass fraction in the hydrophobic sample (8.8 vs 1.3 wt %) inherently decreases the average macroscopic distance between particle domains, the primary driver for the dense nanoscale agglomeration is the loss of this protective coating. This reduction in the organic content suggests a partial loss of the original capping agent. This is further corroborated by FTIR analysis, which indicates that the original ligands have been effectively displaced by the polymer chains, allowing for direct interaction between the iron oxide surface and the PPO/PEO segments. Both favor particle agglomeration, which broadens the size distribution and increases the discrepancy between the Langevin model and experimental measurements. The broader temperature range between the blocking temperature (*T*_b_) and the irreversibility temperature (*T*_irr_) may also result from an increased size distribution caused by the formation of agglomerates as well as enhanced magnetic interactions between nanoparticles within these clusters.

Note that although both hydrophilic and hydrophobic nanoparticles exhibit agglomeration in the matrix, already evidenced by SEM, the superparamagnetic properties of the studied nanocomposites do not only originate from the well-dispersed and isolated IONPs detected by SAXS. As a matter of fact, in superparamagnetic systems, the magnetic moments can rapidly reorient in response to an external magnetic field, preventing long-range magnetic ordering even when particles are aggregated. Aggregation can hinder Brownian relaxation, which relies on the physical rotation of particles, but Néel relaxation, which involves the reorientation of magnetic moments within the particles, remains effective. Despite the reduced mobility due to aggregation, the nanoparticles within the microsized clusters observed in SEM retain their superparamagnetic properties, as the Néel mechanism predominates. This effect is sustained at room temperature, where even with the presence of agglomerates, the clusters remain small enough to stay superparamagnetic. The broad size distribution and resulting overlap of multiple Langevin functions in the MH curve prevent it from displaying a single Langevin behavior.

This was proven by the results in the *χT* plot that shows, even with aggregation and significant size dispersion, that the system maintains a superparamagnetic state at room temperature, allowing nanoparticles to collectively respond to magnetic fields without forming permanent magnetic domains. Furthermore, even for the well-dispersed nanoparticles in the matrix, Brownian relaxation should be inhibited or significantly reduced due to the lower mobility of the nanoparticles compared to particles outside the matrix, due to particle–matrix interactions. This aligns with recent studies on confined magnetic nanomaterials, which demonstrate that Brownian relaxation (physical rotation) is severely constrained or completely suppressed by the high viscosity of the medium and the mechanical coupling between the particles and the matrix.^14^ This also emphasizes the preponderant role of Néel relaxation in these systems.

The heating efficiency of the nanocomposites, quantified by the Specific Absorption Rate (SAR) and Intrinsic Loss Power (ILP), directly reflects the structural and magnetic behaviors elucidated by the previous characterizations. A marked disparity in thermal performance was observed: the hydrophilic samples exhibited superior efficiency (SAR: 70.8–145.3 W/g; ILP: 0.26–0.53 nH·m^2^/kg), whereas the hydrophobic samples presented significantly lower values (SAR: 26.7–48.0 W/g; ILP: 0.10–0.17 nH·m^2^/kg). Variability in the results arises from the spatial distribution of particles, suggesting that the matrix is not perfectly uniform.

These results are in good agreement with values reported in the literature for conventional iron oxide nanoparticles. Notably, the ILP obtained for the nanocomposites loaded with hydrophilic particles (0.26–0.53 nH·m^2^/kg) surpasses some of the used commercial formulations (which exhibit ILP values around 0.16 nH·m^2^/kg, like BNF-02008, or 0.23 nH·m^2^/kg, like Nanomag-D-spio),^45^ indicating a satisfactory conversion efficiency. Although higher values (>5.0 nH·m^2^/kg) are achievable with anisotropic shapes in stable colloids,^46^ the heat generation in the present solid PPO–PEO matrix relies almost exclusively on Néel relaxation (reorientation of the magnetic moment). Brownian relaxation (physical rotation) is severely constrained by the high viscosity of the medium and the mechanical coupling between the particles and the polymer chains. Considering this suppression of the Brownian mechanism, the ILP values achieved by the hydrophilic samples are notable and confirm that the magnetic integrity of the cores was preserved during processing.

The superior performance of the samples containing hydrophilic particles correlates directly with the structural and magnetic findings. As confirmed by FTIR, the weaker interactions between the hydrophilic ligands and the predominantly hydrophobic matrix preserve the particle’s surface functionalization. This prevents the formation of overly dense agglomerates; instead, SEM reveals the presence of well-distributed micrometer-sized clusters, while SAXS analysis simultaneously identifies a diluted system of well-dispersed individual nanoparticles (with an average radius of gyration of 4.3 nm) located in the matrix free volume. Because the nanoparticles are localized in a highly viscous solid matrix, physical rotation (Brownian relaxation) is effectively suppressed, making Néel relaxation the predominant heating mechanism. The structural configuration of the hydrophilic system maintains an optimal “magnetic distance” between the cores, preventing strong dipolar interactions. Consequently, the hydrophilic IONPs preserve a higher saturation magnetization (*M*_s_ = 58.6 emu g^–1^).

Quantitatively, under the low-amplitude alternating magnetic field (AMF) used in this study, the heat dissipation can be described by the Linear Response Theory (LRT), often referred to as the Rosensweig model. In this framework, the Specific Absorption Rate (SAR) is directly proportional to the out-of-phase magnetic susceptibility (χ″), which scales with the square of the saturation magnetization (SAR ∝ *M*_s_^2^). Therefore, the preservation of a high saturation magnetization in the hydrophilic solid nanocomposite disks ensures a strong magnetic response, establishing a high theoretical limit for heat generation.

Furthermore, the efficiency of Néel relaxation depends on the magnetic moments overcoming an energy barrier for magnetization reversal, defined as *E*_a_ = *KV*_eff_ (where *K* is the effective magnetic anisotropy and *V*_eff_ is the effective particle volume). This energy barrier can be semiquantitatively estimated from the blocking temperature (*T*_b_) via the relationship *E*_a_ ≈ 25*k*_B_ *T*_b_. For the hydrophilic nanocomposite (*T*_b_ = 94 K), the energy barrier allows the magnetic moments to efficiently follow the 174.1 kHz AC field. More importantly, the narrow temperature gap between the irreversibility and blocking temperatures (Δ*T* = *T*_irr_ – *T*_b_ = 105 – 94 K = 11 K) is a critical indicator. This narrow Δ*T* demonstrates that the energy barriers, and consequently the particle size distribution and anisotropy contributions, are highly uniform. This structural and magnetic uniformity ensures that a large fraction of the nanoparticles participate coherently in the Néel relaxation process, maximizing the SAR (up to 145.3 W/g).

Conversely, the functional differences in hyperthermia performance for the hydrophobic composites are a direct consequence of their specific surface chemistry. The strong hydrophobic–hydrophobic affinity between the BA-functionalized IONPs and the PPO matrix causes the polymer to displace the original ligands (as indicated by TGA and FTIR). This loss of protective coating significantly impacts their structural configuration, leading to a much denser agglomeration of “naked” strongly interacting cores, as deduced from their spherical morphology in SEM and the significant deviations from the theoretical Langevin model. This dense structural configuration and surface disorder explain the severe approximately 5-fold drop in saturation magnetization (down to 11.8 emu g^–1^). From a physical standpoint, evaluating the Linear Response Theory (LRT) framework (SAR ∝ *M*_s_^2^), this sharp reduction in *M*_s_ inherently limits the capability of the material to absorb magnetic energy. This mathematical scaling justifies the observed 3- to 4-fold decrease in experimental SAR (26.7–48.0 W/g) and ILP (0.10–0.17 nH·m^2^/kg) values compared to the hydrophilic system.

This dense structural configuration also fundamentally alters the Néel relaxation dynamics, which is the almost exclusive heat generation mechanism in this highly viscous solid matrix. The close proximity of the “naked” magnetic cores in the aggregates induces strong interparticle dipole–dipole interactions, which significantly increase the effective magnetic anisotropy (*K*) of the clusters. This increased anisotropy is quantitatively reflected in the higher blocking temperature (*T*_b_ = 103 K), corresponding to a higher overall energy barrier (*E*_a_ ≈ 25*k*_B_ *T*_b_). Crucially, the massive broadening of the gap between the irreversibility (*T*_irr_ = 264 K) and blocking temperatures (Δ*T* = *T*_irr_ – *T*_b_ = 264 – 103 K = 161 K) provides direct evidence of a highly heterogeneous system. This wide Δ*T* clarifies that the broad size distribution of the aggregate clusters creates a massively broadened distribution of energy barriers and anisotropy contributions. Because these strongly interacting clusters possess energy barriers that are too widely distributed and often too high, a significant portion of their magnetic moments cannot effectively align and reverse with the rapid oscillation of the applied AC field. As a result, the Néel relaxation is severely suppressed, preventing efficient heat dissipation in the solid matrix.

In summary, while chemical compatibility (hydrophobic–hydrophobic) is generally desired for structural homogeneity, in the context of magnetic hyperthermia, it paradoxically led to “overinteraction” and aggregation that proved detrimental to the specific loss of power. The hydrophilic functionalization offered a better balance, maintaining a “magnetic distance” between cores that favors efficient energy dissipation via Néel relaxation, consistent with the theoretical framework where reduced aggregation preserves a higher average magnetic moment.^47^ These findings align with recent studies that optimizing surface functionalization and preventing agglomeration remain the key frontiers for advancing magnetic hyperthermia.^48,49^ While the severe aggregation and the resulting strong dipolar interactions are primary drivers for the hindered Néel relaxation, it is important to acknowledge that other intrinsic factors related to the altered surface chemistry may also contribute to the reduced hyperthermia performance. For instance, the displacement of the organic ligands might induce surface spin disorder (spin canting), intrinsically lowering the magnetic moment of the individual cores. Additionally, the direct contact between the “naked” nanoparticle and the polymer might alter the interfacial thermal transfer compared to a fully passivated surface. However, fully disentangling the exact quantitative contribution of these intrinsic boundary effects from the secondary aggregation effects is beyond the scope of this study, which focuses on the pronounced structural and macro-magnetic consequences of the modified surface chemistry.

## Conclusions

This study demonstrated that controlling the surface chemistry of superparamagnetic iron oxide nanoparticles is of fundamental importance for optimizing the magnetic hyperthermia performance of Siloxane-PPO-PEO solid nanocomposite disks. By comprehensively integrating multiscale structural and magnetic characterizations, a clear structure–property-function relationship was established. Surprisingly, a strong chemical affinity between the highly hydrophobic BA-functionalized nanoparticles and the predominantly hydrophobic matrix led to the displacement of the protective organic coating. This disruption caused the “naked” magnetic cores to densely aggregate at the nanoscale. Consequently, strong dipolar and exchange interactions within these dense clusters drastically reduced the saturation magnetization to 11.8 emu/g and broadened the *T*_b_ – *T*_irr_ gap. From a functional standpoint, this suppressed magnetic moment hindered the Néel relaxation process, resulting in poor heating efficiency, with SAR values limited to 26.7–48.0 W/g.

Conversely, the weaker interactions between the hydrophilic TEG-functionalized nanoparticles and the matrix successfully preserved their surface coating. This structural preservation maintained an optimal magnetic distance between nanoparticles. As a result, the hydrophilic system retained a high saturation magnetization (58.6 emu/g), maximizing Néel relaxation and yielding SAR and ILP values up to 3 to 4 times higher than the hydrophobic system. Overall, these findings highlight that preserving nanoparticle coating to prevent dense nanoscale aggregation is critical for maintaining the high effective magnetic moments required for efficient magnetic hyperthermia in solid nanocomposite disks.

Further investigations are in course aiming to modulate the particle–matrix affinity through both the adequate choice of chemical groups for particle covering and the variations of the proportion between hydrophilic PEO and hydrophobic PPO in the matrix. This perspective, combined with the possibility of inducing drug delivery by disrupting the hydrogen bonds between urea groups due to thermal expansion promoted by magnetic hyperthermia, highlights the potential of siloxane-polyether hybrids as innovative therapeutic materials.

## Supplementary Material



## Notes

Additional characterization techniques used to analyze both the iron oxide nanoparticles and the siloxane-polyether hybrid materials with and without nanoparticles. Thermogravimetric analysis (TGA) was performed to assess the thermal stability and decomposition behavior of the hybrid materials. X-ray diffraction (XRD) was used to confirm the crystalline structure of the nanoparticles. Vibrating sample magnetometry (VSM) provided insights into the magnetic properties and interactions of the nanoparticles. These characterizations serve as a basis for comparison between the nanoparticles, the pure polymer, and the nanocomposites, helping to understand the impact of nanoparticle incorporation. Detailed statistical information regarding the SAXS data fitting (PDF)
