# Supplementary material for: Siloxane-PEO-PPO Hybrid Materials Containing Superparamagnetic Iron Oxide Nanoparticles: Effect of Particle Surface Functionalization on the Structure and Hyperthermia Properties
Source: ACS Omega. 2026 Jun 6;11(24):35434–49. doi: 10.1021/acsomega.6c01026 (PMC13295056; doi:10.1021/acsomega.6c01026)
Supplement: Supplementary file 1 [file ao6c01026_si_001.pdf]

Title Page

Siloxane-PEO-PPO hybrid materials containing Superparamagnetic Iron Oxide Nanoparticles: effect of Particles Surface Functionalization on Structure and Hyperthermia Properties

Agnes Candido Teixeira[1]*, *Natasha Midori Suguihiro*[1], *Benjamin Rache Salles*[2], *Pedro Carvalho Ramos*[1], *Luiz Augusto Sousa de Oliveira*[1], *Karim Dahmouche*[1]

[1]Campus de Duque de Caxias, Universidade Federal do Rio de Janeiro (UFRJ) - Duque de Caxias, RJ, Brazil CEP 25240-000

[2]Instituto de Física, Universidade Federal do Rio de Janeiro (UFRJ) - Rio de Janeiro, RJ, Brazil CEP 21941-909

* Corresponding Author
e-mail: agnescteixeira@gmail.com
phone number: +55 21 988903510

Supporting Information

Thermogravimetric Analysis.

Thermogravimetric Analysis (TGA) was performed using a Q500 thermogravimetric analyzer (TA Instruments) under a nitrogen atmosphere with a heating rate of 10 °C/min from room temperature up to 700 °C. The thermogravimetric analysis (TGA) of the hybrid material without nanoparticles showed distinct mass loss stages, corresponding to the decomposition of its components (Figure. S1).

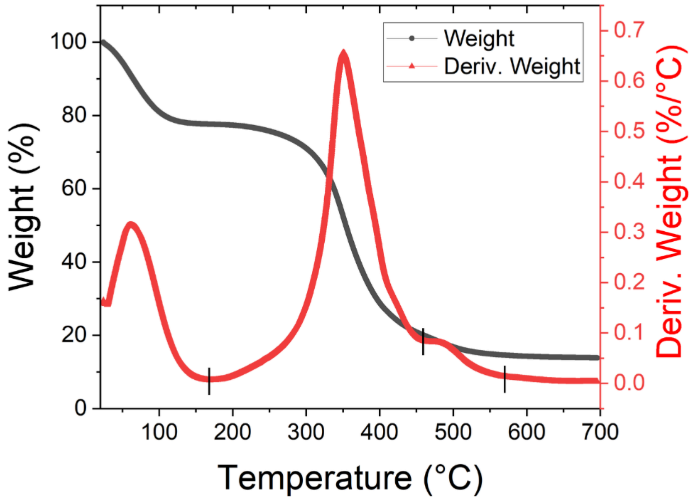

Figure S1. Thermogravimetric Analysis (TGA) profiles of Siloxane-PPO-PEO hybrid.

For the nanocomposites containing hydrophilic and hydrophobic iron oxide nanoparticles within the Siloxane-PPO-PEO matrix, the TGA revealed additional mass loss stages, reflecting the contributions of the nanoparticles (Figure. S2). Tables S1 and S2 summarizes the main events detected for the studied hybrid materials.

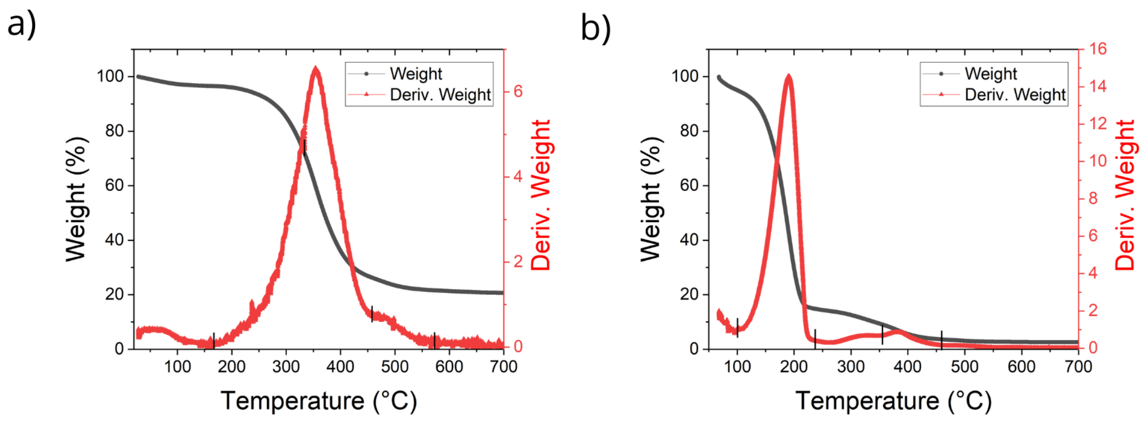

Figure S2. Thermogravimetric analysis (TGA) of (a) siloxane-PPO-PEO−IONPs nanocomposite with hydrophobic nanoparticles and (b) siloxane-PPO-PEO−IONPs nanocomposite with hydrophilic nanoparticles.

Table S1. TGA events for Siloxane-PPO-PEO hybrids with and without Fe_2O_3 nanoparticles.

Sample	Temperature Range (°C)	Thermal Event	Mass loss (%)
Siloxane-PPO-PEO	Room temp - 156°C	Evaporation of water and ethanol	22.3%
	156°C - 460°C	Polymer degradation	58%
	460°C - 569°C	Decomposition of non-hydrolyzed ethoxy groups	5.4%
	Residual mass	Inorganic siloxane phase	13.8%
Siloxane-PPO-PEO−IONPs (Hydrophobic IONPs)	Room temp - 169°C	Evaporation of water and ethanol	3.5%
	169°C - 460°C	Decomposition of polymer content and organic solvent	70.2% (24.6% polymer, 45.6% solvent)
	460°C - 569°C	Decomposition of non-hydrolyzed ethoxy groups	4.7%
	Residual mass	Inorganic siloxane phase	11.2%
	Residual mass	Iron oxide nanoparticles	8.8%

Siloxane-PPO-PEO−IONPs (Hydrophilic IONPs)	Room temp - 102°C	Evaporation of water and ethanol	5.1%
	102 °C – 236.4 °C	Decomposition of organic solvent	80 %
	236.4°C - 460°C	Polymer degradation	5.7%
	Residual mass	Silica and nanoparticles	1.3%
	Residual mass	Iron oxide nanoparticles	1.3 %

Table S2. TGA events for Siloxane-PPO-PEO hybrids with and without Fe_2O_3 nanoparticles by subtracting the contribution of water, ethanol and organic ligands located at the particle's surface.

Sample	Temperature Range (°C)	Thermal Event	Mass Loss (%)
Siloxane-PPO-PEO	156°C - 460°C	Decomposition of polymer content	75 %
	460°C - 569°C	Decomposition of unhydrolyzed ethoxy groups	7 %
	Residual mass	Inorganic silica phase	18 %
Siloxane-PPO-PEO−IONPs (Hydrophobic IONPs)	169°C - 460°C	Decomposition of polymer content	49.8 %
	460°C - 569°C	Decomposition of unhydrolyzed ethoxy groups	9.6 %

	Residual mass	Inorganic silica phase	22.8 %
	Residual mass	Iron oxide nanoparticles	17.8%
siloxane-PPO-PEO−IONPs (Hydrophilic IONPs)	236.43°C - 460°C	Decomposition of polymer content	68,9 %
	Residual mass	Inorganic silica phase	16 %
	Residual mass	Iron oxide nanoparticles	15,1%

X-ray Diffraction (XRD).

The X-ray diffraction (XRD) patterns obtained for both hydrophilic and hydrophobic iron oxide nanoparticles revealed characteristic diffraction peaks, consistent with those reported in the literature for iron oxides. Specifically, the peaks observed align with the known diffraction patterns for magnetite (Fe_3O_4) and maghemite (γ-Fe_2O_3) (Figure. S3). For maghemite, the common peaks appear at 2θ values of 30.2°, 35.6°, 43.3°, 53.7°, 57.3°, and 62.9°, corresponding to the (220), (311), (400), (422), (511), and (440) crystallographic planes, respectively. For magnetite, these peaks are observed at slightly different 2θ values: 30.1°, 35.4°, 43.2°, 53.5°, 56.9°, and 62.5°[41,42].

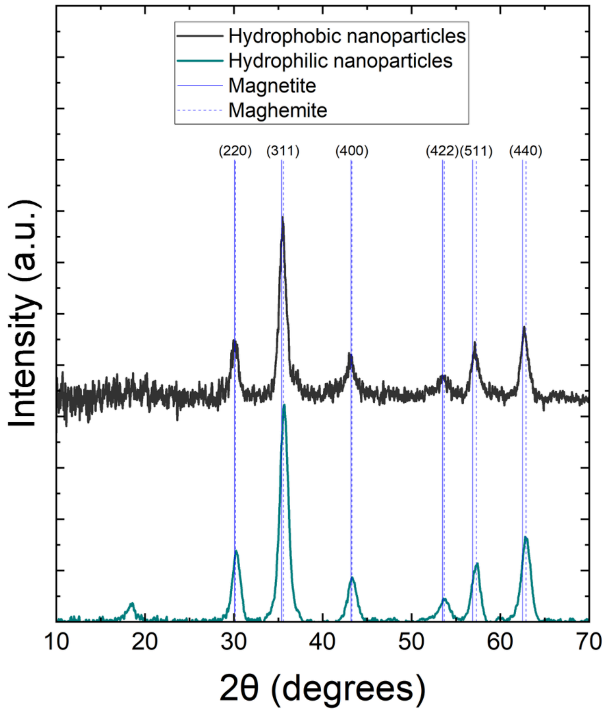

Figure S3. X-ray diffraction (XRD) patterns of hydrophilic and hydrophobic iron oxide nanoparticles with reference peaks for magnetite (Fe_3O_4) and maghemite (γ-Fe_2O_3).

Vibrating Sample Magnetometer (VSM).

The magnetization curves M(H) at 300 K and M(T) at 20 Oe for colloidal suspensions of hydrophilic and hydrophobic iron oxide nanoparticles were analyzed in detail.

For the hydrophilic nanoparticles, the blocking temperature (Tb) was observed at 66K, while the irreversibility temperature (Tirr) was 71K (Figure. S4a). These temperatures are close to each other, suggesting weak magnetic interactions between the nanoparticles and a narrow size distribution. The proximity of Tb and Tirr indicates that the system has minimal interaction and good nanoparticle coating. Below Tb, the relaxation is slow, and the magnetic moment is difficult to reorient, while above Tb, the system behaves more like a paramagnet with rapid relaxation. Tirr is calculated when the difference between the ZFC (Zero Field Cooled) and FC (Field Cooled) curves is around 1%.

For the hydrophobic nanoparticles, the Tb was observed at 236K, while the Tirr was 322K (Figure. S4d). These temperatures, being higher than those of the hydrophilic nanoparticles, suggest a similar behavior but at a higher temperature range. Note that ZFC and FC curves converge to one curve at temperature below the higher measurement temperature and thus validating the analysis of the temperature dependence of the magnetization. The larger difference between Tb and Tirr reveals stronger magnetic interactions, as expected for IONPs powder of larger particles. Still, this observation is in agreement with a good nanoparticle coating. The behavior of the magnetic moments above Tb aligns with the expected rapid relaxation similar to a paramagnetic system, while below Tb, the blocked state indicates a slow relaxation.

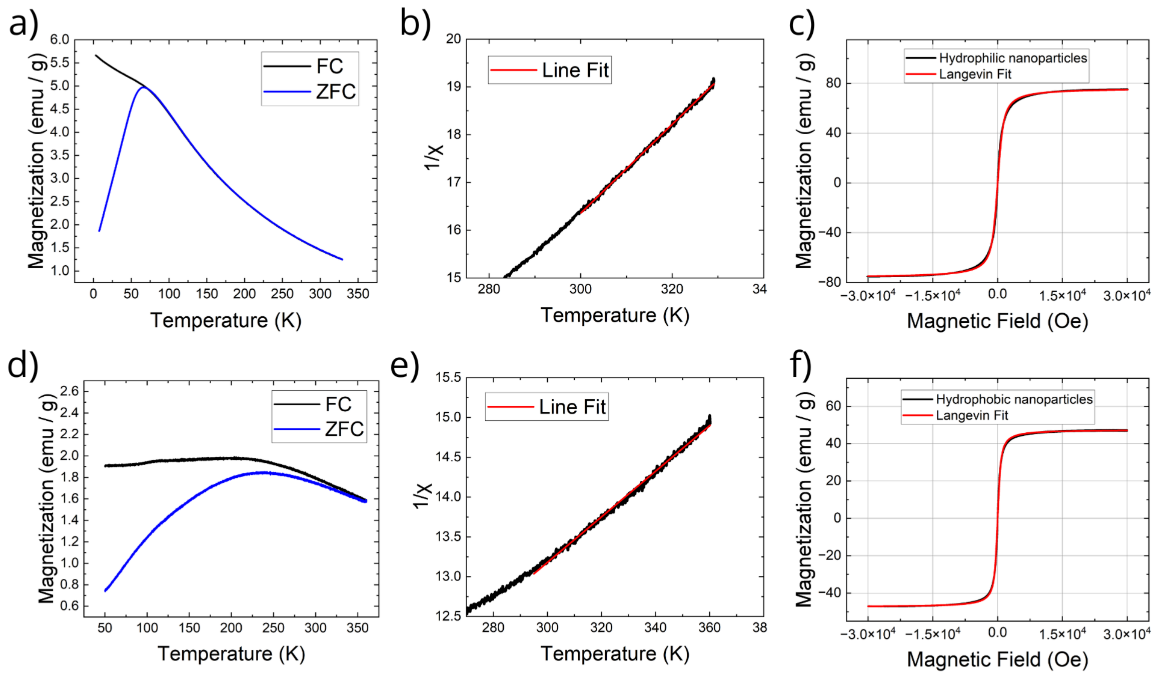

Figure S4. Magnetic characterization of hydrophilic and hydrophobic iron oxide nanoparticles. (a) ZFC and FC magnetization curves for hydrophilic nanoparticles as a function of temperature. The blocking temperature (Tb) is observed at 66K, and the irreversibility temperature (Tirr) is at 71K. (b) Inverse magnetic susceptibility (1/χ) versus temperature for hydrophilic nanoparticles, showing linear behavior above 300K. (c) Magnetization curve for hydrophilic nanoparticles at 300K fitted with the Langevin function, resulting in a saturation magnetization of 76 emu/g and an average magnetic moment of 2.6 x 10^{-19} emu. (d) ZFC and FC magnetization curves for hydrophobic nanoparticles as a function of temperature. The blocking temperature (Tb) is observed at 236K, and the irreversibility temperature (Tirr) is at 322K. (e) Inverse magnetic susceptibility (1/χ) versus temperature for hydrophobic nanoparticles, showing linear behavior above 300K. (f) Magnetization curve for hydrophobic nanoparticles at 300K fitted with the Langevin function, resulting in a saturation magnetization of 47 emu/g and an average magnetic moment of 4.8 x 10^{-19} emu.

To better observe the transition to the superparamagnetic regime, we plotted the inverse magnetic susceptibility (1/χ) as a function of temperature (Figure. S4b and S4e). The linearity of 1/χ confirms the superparamagnetic behavior of the samples. The Curie constant (C) and Weiss temperature (ΘCW) were also determined for both types of nanoparticles. For the hydrophilic nanoparticles, the Curie constant (C) was calculated as 10.75 emu/Oe.K, with a Weiss temperature (ΘCW) of 124.11K. For the hydrophobic nanoparticles, the Curie constant (C) was 34.94 emu/Oe.K, with a Weiss temperature (ΘCW) of -160.46K. These

values were derived from the linear relationship of the inverse magnetic susceptibility ($1/\chi$) as a function of temperature, which follows the Curie-Weiss law: $\chi = C/(T\text{-}\Theta CW)$. The positive Weiss temperature for hydrophilic nanoparticles indicates ferromagnetic interactions, while the negative value for hydrophobic nanoparticles suggests antiferromagnetic interactions.

The Langevin function was used to fit the magnetization curves at 300K. For the hydrophilic nanoparticles, the fit resulted in a saturation magnetization of 76 emu/g and an average magnetic moment per nanoparticle of 2.6 x 10^{-19} emu (Figure. S4c). For the hydrophobic nanoparticles, the fit yielded a saturation magnetization of 47 emu/g and an average magnetic moment per nanoparticle of 4.8 x 10^{-19} emu (Figure. S4f). In a few instances, the Langevin function did not perfectly fit the field-dependent behavior, likely due to the size distribution of the particles, but this did not affect the linear behavior of $1/\chi$ in the superparamagnetic regime. These detailed analyses of the magnetic properties of hydrophilic and hydrophobic nanoparticles provide valuable insights into their interactions and behavior, essential for optimizing their applications in superparamagnetic systems.

Small-Angle X-Ray Scattering (SAXS)

The tables provided in this section contain the numerical results of the non-linear regression analysis conducted via Origin software. Statistical indicators, such as the R-squared values and standard errors, are included for each fit. The Rg values are expressed in Angstroms since the fits of the SAXS curves have been done with q-values expressed in A^{-1}. The q-values have been then converted in nm^{-1} for presentation in figure 7 of the manuscript. The parameter named G in the fit (from "Guinier") is the parameter I(0) in the manuscript.

Statistics

	log I(q)
Number of Points	17
Degrees of Freedom	15
Reduced Chi-Sqr	10749,37408
Residual Sum of Squares	161240,61115
R-Square (COD)	0,99618
Adj. R-Square	0,99592
Fit Status	Succeeded(100)

Fit Status Code :
100 : Fit converged. Chi-Sqr tolerance value of 1E-9 was reached.

Summary

	G		Rg		Statistics	
	Value	Standard Error	Value	Standard Error	Reduced Chi-Sqr	Adj. R-Square
log	11850,18067	151,70475	42,62551	0,37173	10749,37408	0,99592

Figure S5. SAXS data fitting parameters and statistical indicators for the sample loaded with the hydrophobic iron-oxide particles.

Statistics

	log I(q)
Number of Points	15
Degrees of Freedom	13
Reduced Chi-Sqr	1049,15512
Residual Sum of Squares	13639,01653
R-Square (COD)	0,99737
Adj. R-Square	0,99717
Fit Status	Succeeded(101)

Fit Status Code :
101 : Fit converged - Chi-sqr no longer changed.

Summary

	G		Rg		Statistics	
	Value	Standard Error	Value	Standard Error	Reduced Chi-Sqr	Adj. R-Square
log I(q)	4844,18137	52,94856	43,1426	0,33	1049,15512	0,99717

Figure S6. SAXS data fitting parameters and statistical indicators for the sample loaded with the hydrophilic iron-oxide particles.
